# Proceedings of 6th European Dirofilaria and Angiostrongylus Days: Belgrade, Serbia. July 5 - 7, 2018

**DOI:** 10.1186/s13071-018-3183-z

**Published:** 2018-12-12

## Invited Presentations

### I1. *Dirofilaria* infections in Europe: a short update

#### Genchi C. (claudio.genchi@unimi.it)

##### Department of Veterinary Medicine, University of Milan, Milan, Italy

Currently, *Dirofilaria* infections are endemic in all over Europe, particularly *D. repens* which is spreading from Spain until North East Siberia (Yakutsk 62°N, 130°E) and it has been reported at northern limit of 62°N, 41°E (Arkhagelsk, North of Russia). While the cases of canine *D. immitis* infections seem quite limited, probably because if they are not cured the disease become more and more severe, the number of *D. repens* infections both in dogs and in humans are continuously increasing. It must to be emphasized that most of *D. repens* infections in dogs, which are the most important reservoir, are completely asymptomatic and can be diagnosed only by the visualization of circulating microfilariae in the blood. So, the only way to decrease the spreading of infection and to protect the human health is to control all the dogs in endemic areas and treating them against the infection.

### I2. Current situation of animal and human dirofilariosis in Iberian Peninsula

#### Morchón R.^1^, Carretón E.^2^, Zueva T,^1^, Diosdado A.^1^, Sed G.^1,3^, Kartashev V.^4^, González-Miguel J.^5^, Simón F.^1^

##### ^1^Laboratory of Parasitology, Animal and human dirofilariosis group, Faculty of Pharmacy, University of Salamanca, Campus Miguel Unamuno s/n, 37007 Salamanca, Spain; ^2^Research Institute of Biomedical and Health Sciences (IUIBS), University of Las Palmas de Gran Canaria, 35001 Las Palmas, Spain; ^3^Department of Veterinary Science, University of Pisa, Viale delle Piagge 2, 56124 Pisa, Italy; ^4^Rostov State Medical University, Rostov-na-Donu, 344022, Russia; ^5^Institute of Natural Resources and Agrobiology of Salamanca (IRNASA-CSIC), 37008, Salamanca, Spain

###### **Correspondence:** Morchón R. (rmorgar@usal.es)

Most of the available epidemiological information on animal and human dirofilariosis originates from a limited number of countries in which this parasitosis has been considered as both a veterinary and medical concern for decades. Nevertheless, with increasing frequency, data are being generated in countries where dirofilariasis was not previously documented or for which information was limited. Several factors can exert an influence on the spreading of the disease, such as movement of infected animals, the introduction of new species of mosquitoes able to act as vectors, the climate change caused by the global warming, and development of human activity in new areas. The Iberian Peninsula is a territory with a wide variety of climates: Oceanic, Mediterranean and Continental. There are also rugged and varied relief with mountain ranges, wide plateaus and narrow coastal strips, which originate a complicated distribution of clouds, insolation, rain and winds. To this we must add the extension of irrigated areas directly influences the presence of dirofilariosis in a certain place. Although the Iberian Peninsula is considered an endemic area, not all of the territory has been sampled. From what is known, the highest prevalences are still in the southern in the coastal areas and in the irrigated areas and near the rivers. In addition, canine dirofilariosis is present in Madeira Island and Canary Islands but, in these cases, only the presence of *Dirofilaria immitis* has been reported so far. Regarding feline dirofilariasis, only seroepidemiological studies carried on in Canary Islands, Barcelona, Madrid and in the central and northern Portugal showed an increase in prevalences. There are no new data on human dirofilariosis with the exception of two recently imported cases. In wild animals there is no new case or study reported. As regards *Dirofilaria* vectors, two species responsible for transmission have been reported, such as *Culex pipiens* and *Cx. theileri*. Finally, appliying Geographic Information Systems (GIS) has been possible to predict the distribution of the risk of transmission of dirofilariosis and the changes in the Iberian Peninsula, based on temperatures and rainfall data as well as on the distribution of irrigated crops areas. Highest transmission risk exists in several areas where moderate/high temperatures coincide with extensive irrigated crops.

### I3. Mosquito vectors of *Dirofilaria* spp. and the situation of *Dirofilaria* spp. in Central Europe

#### Fuehrer H. P. (hans-peter.fuehrer@vetmeduni.ac.at)

##### Institute of Parasitology, Department of Pathobiology, University of Veterinary Medicine, Vienna, Austria

*Dirofilaria immitis* and *D. repens* are filarioid helminths with domestic and wild canids as main hosts and mosquitoes as vectors. Both species are known to be zoonotic. *Dirofilaria repens* and *D. immitis* are associated with climate change and a spread from historically endemic countries in Southern Europe to Central Europa has been observed. Until very recently both species were known not to be endemic in Austria [1]. Several xeno-monitoring studies were conducted to evaluate the presence of filarioid mosquitoes [2-3]. Although more than 70,000 mosquitoes were screened in the past years, DNA of *D. repens* was only found in *Anopheles algeriensis*, *An. plumbeus* and the *An. maculipennis*-complex collected in Eastern Austria close to the border to Slovakia and Hungary – two countries where both *D. repens* and *D. immitis* are known to be endemic. Most of the previous studies evaluated the presence of filarioid helminths in mosquitoes using xeno-monitoring tools (e.g. pools, use of entire mosquitoes). Lab-based vector competence studies or other vector competence studies have been conducted only rarely [4-5]. An overview on the current situation of *Dirofilaria* spp. in Austria and neighboring countries will be given and causatives for differences in prevalence discussed. Moreover, the benefits but also risks (e.g. interpretation) of mosquito xeno-monitoring studies will be presented.

References

1. Fuehrer HP, Auer H, Leschnik M, Silbermayr K, Duscher G, Joachim A. *Dirofilaria* in humans, dogs, and vectors in Austria (1978-2014) - from imported pathogens to the endemicity of *Dirofilaria repens*. PLoS Negl Trop Dis. 2016;10:e0004547.

2. Silbermayr K, Eigner B, Joachim A, Duscher GG, Seidel B, Allerberger F, et al. Autochthonous *Dirofilaria repens* in Austria. Parasit Vectors. 2014;7:226.

3. Übleis SS, Cuk C, Nawratil M, Butter J, Schoener E, Obwaller AG, et al. Xenomonitoring of mosquitoes (Diptera: Culicidae) for the presence of filarioid helminths in eastern Austria. Can J Infect Dis Med Microbiol. 2018; ID 9754695 (In Press).

4. Silaghi C, Beck R, Capelli G, Montarsi F, Mathis A. Development of *Dirofilaria immitis* and *Dirofilaria repens* in *Aedes japonicus* and *Aedes geniculatus*. Parasit Vectors. 2017;10:94.

5. Ionică AM, Zittra C, Wimmer V, Leitner N, Votýpka J, Modrý D, et al. Mosquitoes in the Danube Delta: searching for vectors of filarioid helminths and avian malaria. Parasit Vectors. 2017;10:324.

### I4. Emerging human dirofilariasis in the former USSR

#### Simón F.^1^, Kurpita V.^2^, Nikolaenko S.^2^, Matyushkina K.^2^, Sagach O.^2^, Korzan A.^3^, Yashkova S.^3^, Guzeeva T.^4^, Shapiyeva Z.^5^, Getpisbaeva G.^5^, Zumbulidze N.^6^, Ilyasov B.^7^, Bastrikov N.^8^, Ambalov Y.^8^, González-Miguel J.^1^, Morchón R.^1^, Siles-Lucas M.^9^, Kartashev V.^8^

##### ^1^Laboratory of Parasitology, Animal and human dirofilariosis group, Faculty of Pharmacy, University of Salamanca, Campus Miguel Unamuno s/n, Salamanca, Spain; ^2^The Public Health Center of the Ministry of Health of Ukraine, Kiev, 04071, Ukraine; ^3^Republican Center of Hygiene, Epidemiology and Public Health, Minsk, Belarus; ^4^Sechenov First Moscow State Medical University, Moscow 119991, Russia; ^5^Ministry of Health Republic of Kazakhstan, Almaty, 050008, Kazakhstan; ^6^Mechnikov North-Western State Medical University, Saint-Petersburg, 191015, Russia; ^7^Rostov Oblast Medical Diagnostic Center, Rostov-na-Donu, 344010, Russia; ^8^Rostov State Medical University, Rostov-na-Donu, 344022, Russia; ^9^Instituto de Recursos Naturales y Agrobiología, CSIC, Salamanca, Spain

###### **Correspondence:** Simón F. (fersimon@usal.es)

Human dirofilariosis (HD) emerged 20 years ago when from a casuistic disease it became a real medical problem. That time HD was most often registered at the southern part of Europe including the territory of the former USSR. HD emergence switched case report studies to systematic research. Ukrainian parasitologists introduced well managed system of notification and centralized reference laboratory confirmation of each HD case in 1997. It explains why the highest number of HD cases was reported from there (2184). In Russia HD became a notifiable disease from 2014 (1846 cases from 1997). In Belorussia 62% of all HD cases were registered in 2012-2017 (out of 134 from 1997). Fewer HD patients were reported from Kazakhstan (22 from 1997). Seroepidemiology study of 318 people established that 10% of healthy population was at risk of infection at the south-western part of Russia. Our spatiotemporal GIS model elaborated in 2012 correctly predicted future expansion of HD to the North especially extensive in the European part of the former USSR territory. New HD cases are now reported up to the 61° nl in western Russia. Autochthonous HD cases were recently diagnosed in Baltic republics and in southern Finland bordered with Russia. Based on 944 patients’ records we have analyzed HD clinical presentation. Our results showed that HD patients are mostly 21 - 60 years old (70%), women are much more often affected (72%) than men. Eye area (39%) followed by head and neck (31%), then limbs (20%) and trunk (5%), and rarely men genitalia (3%) and women breast (2%) are the sites of HD parasite location. HD is not well known to medical doctors especially in the countries where the disease is rare or presented as imported cases. Based on our focused study we demonstrate that the only way to suspect a well-grounded pre-surgery diagnosis is an ultrasound examination including color Doppler charting that allows finding some well-defined characteristics that are specific for HD. DNA amplification of specific 12S rRNA subunit of the excised parasites followed by sequences based phylogenetic analysis revealed that 14% of HD ocular cases were caused by *Dirofilaria immitis*. The emergence of HD in Europe, the established role of *D. immitis* in some of HD ocular cases, the high seroprevalence in healthy population should stimulate interdisciplinary and international research of HD aiming to prevent growing morbidity and its further spread.

### I5. Are all MLs the same when combined with doxycycline for adulticide therapy?

#### Kramer L. H. (kramerlh@unipr.it)

##### Department of Veterinary Sciences, University of Parma, Parma 43126, Italy

Heartworm infection (*Dirofilaria immitis*) in dogs causes chronic pulmonary disease that, if left untreated, can lead to right-side congestive heart failure. Currently, the only registered drug for adulticide therapy in dogs with heartworm disease (HWD) is melarsomine dihydrochloride (Immiticide®, Merial). The recent targeting of the bacterial endosymbiont *Wolbachia*, through antibiotic therapy of the infected host, has offered an interesting alternative for the treatment of HWD. *Wolbachia* is necessary for the reproductive capacity and long-term survival of those filarial parasites that harbour the endosymbiont. Recent reports of the adulticide activity of an ivermectin/doxycycline combination protocol has led the American Heartworm Society (AHS) to include in its guidelines that in cases where arsenical therapy is not possible or is contraindicated, a monthly heartworm preventive along with doxycycline for a 4-week period might be considered. Twenty dogs with natural *D. immitis* infection were treated with a combination of doxycycline (10 mg/kg/bid) for 30 days together with a topical formulation containing 10 % w/v imidacloprid and 2.5 % w/v moxidectin (Advocate®, Bayer Health Care, Germany) monthly for nine months, following owners’ consent. Knott and antigen testing was carried out monthly, cardiac ultrasound and thoracic radiographs were performed at enrolment and at 6 and 9 months and scored. Monthly moxidectin (for 9 months) combined with 30 days of doxycycline eliminated circulating microfilariae within one month, thus breaking the transmission cycle very quickly. Furthermore, dogs started to become negative for circulating antigens at 4 months from the beginning of treatment and were all antigen negative at 9 months. No dogs showed worsening of Eco/thoracic Rx scores. When compared to data for doxycycline combined with ivermectin, dogs treated with moxidectin and doxycycline become negative for microfilariae and antigens much sooner and also in this case, show no worsening of clinical signs. Combinations of macrocyclic lactones and doxycycline are safe and effective adulticde alternatives for heartworm disease and moxidectin is more efficient in the combination protocol compared to ivermectin.

This study was supported by Bayer Animal Health.

### I6. Slow-kill protocols – what works, what doesn’t

#### Rishniw M. (mr89@cornell.edu)

##### BVSc, MS, PhD, Dip. ACVIM (Internal Medicine and Cardiology) Cornell University USA

Over the last 10 years, investigators have proposed protocols other than arsenical administration as an alternative way of eliminating adult *Dirofilaria immitis*. These protocols mostly consist of combinations of macrocyclic lactones and tetracycline derivatives. However, questions remain about the efficacy of these approaches and concerns about enabling development of resistance. This talk will discuss the various approaches, results and potential complications of slow-kill adulticide therapy.

### I7. Slow-kill protocols – what works, what doesn’t

#### Rishniw M. (mr89@cornell.edu)

##### BVSc, MS, PhD, Dip. ACVIM (Internal Medicine and Cardiology) Cornell University USA

Since 2005, practitioners in parts of the United States have expressed concerns about “loss of efficacy” of heartworm preventatives, arguing that resistance to these drugs is developing. Several studies have examined the issues of resistance, but the experts are divided about whether it is a real problem, an emerging concern that will affect worldwide efficacy of heartworm preventatives, or whether it is all due to human error. This talk will examine the issue of heartworm resistance and provide arguments for and against the issue.

### I8. Dilemmas of heartworm disease

#### Rishniw M. (mr89@cornell.edu)

##### BVSc, MS, PhD, Dip. ACVIM (Internal Medicine and Cardiology) Cornell University USA

Although heartworm disease has been studied for 50 years, some situations remain largely unaddressed. What do I do about discordant test results? Is “HARD” (Heartworm Associated Respiratory Disease) in cats a real phenomenon? My dog exceeds the weight limit for injections of melarsomine – what do I do? What methods exist for identifying microfilaria? What do I tell clients who occasionally take their pets to endemic areas? Do I need to kill microfilariae acutely after adulticide therapy? Why did my melarsomine treatment fail? This talk will cover multiple “tid-bits” that can help clinicians better understand the biology of heartworm disease.

### I9. Pharmacological management of PHT and right congestive failure

#### Venco L. (luigivenco@libero.it)

##### Clinica veterinaria Lago Maggiore, Arona, Italy

Given the disease pathogenesis, Pulmonary Hypertension (PHT) in Heartworm infected dogs should be considered as a Type 1 Hypertension (Pulmonary arterial hypertension) due to proliferative endarteritis and anatomical damage of the vessels that can be complicated by or develop together with thromboembolism following the death of worms, leading to Type 4 Hypertension (Chronic thromboembolic pulmonary hypertension) [1]. Different drugs can lead therefore to different results based on the prevalent mechanism causing PHT. Besides removing/eliminating the cause (the adult heartworms) also considering that an adulticide therapy could aggravate thromboembolism and that the elimination of parasites prevents the worsening of the clinical situation but often in advanced stages does not lead to a significant improvement, collateral therapy can be useful. At the moment forced rest appears the most important aid, but phosphodiesterase 5 inhibitors when vasoconstriction is present and corticosteroids if significant pulmonary and perivascular inflammation is present may give some results. In case of right heart congestive failure with abdominal effusion, diuretics (torsemide appears probably more appropriate than fursemide) are the mainstay of therapy and they should be used at the lowest dose possible to control the symptoms together with drugs and dietary management useful to counter cachexia following heart failure.

References

1. Simonneau G, Gatzoulis MA, Adatia I, Celermajer D, Denton C, Ghofrani A, et al. Clinical classification of pulmonary hypertension. J Am Coll Cardiol. 2013;62(Suppl. 25):D34-41.

### I10. Diagnosis of feline heartworm disease

#### Manzocchi S. (Simone.Manzocchi@gmail.com)

##### Novara Day Lab, IDEXX Laboratories, Italy, Granozzo con Monticello 28060

Diagnosis of heartworm infection in the cats is extremely challenging. The different biological features of the parasite, elusive clinical picture and peculiar physiopathology of the infection in the cat compared to the dog make a multistep diagnostic approach mandatory. Furthermore, most of the diagnostic techniques that have high clinical utility, sensitivity and specificity in the dog have a worse diagnostic performance in cats. At present time there is no single ante mortem diagnostic test that can reach high levels of sensitivity for feline heartworm infection, especially considering the unique adult- and premature-stage associated syndromes. A holistic approach and careful interpretation of the results of several tests are required for achieving an accurate diagnosis of feline heartworm disease. Thoracic radiography and serum antibody tests are useful for rising the index of suspicion for the disease, while echocardiography, serum antigen tests and tests for concentration and identification of circulating microfilariae are the only ones that can confirm the infection. Other tests, like complete blood count or bronchoalveolar lavage, should be considered of secondary importance, even if they can help supporting the clinical suspicion. Differently from canine infection, microfilaremia is rarely observed in the cat and cannot be considered a sensitive test for diagnosis of *D. immitis*, however, when observable, specificity is 100%, as in dogs. Tests detecting adult heartworm antigens are very specific and can provide a definitive proof of infections in cats, but their sensitivity can be very low considering the small number of adults that develop in cats and the possibility of clinical disease due to immature worms. Heat treatment of serum samples may increase the sensitivity of antigen tests in cats by disrupting immune complexes (Antigen masking). Tests for detection of antibodies to heartworm can be useful to decrease the index of suspicion and asses the risk of exposition. In case of infection and clinical disease caused by premature stages, antibodies tests can have a specific clinical utility, while antigen tests and microfilaremia are generally negative. Antibody tests can have variable sensitivity based on the chosen cut-off of positivity, but generally the specificity is moderate due to cross reactions with other parasites and persistence of antibodies in abortive infections. Sensitivity can be high in recent infections and exposition, but generally decreases with the time during infection.

### I11. Cardiopulmonary biomarkers: how useful are they for the assessment, treatment, and prognosis of heartworm disease?

#### Carretón E.^1^, Morchón R.^2^, Falcón-Cordón Y.^1^, Falcón-Cordón S.^1^, Montoya-Alonso J. A.^1^

##### ^1^Internal Medicine, Faculty of Veterinary Medicine, Research Institute of Biomedical and Health Sciences (IUIBS), University of Las Palmas de Gran Canaria, 35411, Spain; ^2^Laboratory of Parasitology, Group of animal and human dirofilariosis, Faculty of Pharmacy, University of Salamanca, Campus Miguel Unamuno s/n, 37007, Spain

###### **Correspondence:** Carretón E. (elena.carreton@ulpgc.es)

Cardiopulmonary biomarkers are serum parameters that can be objectively measured and quantified as indicators of pathogenic processes, and are considered tools for the screening, diagnosis or monitoring of disease, and determining the prognosis. They may also be used to determine the usefulness of specific therapies and susceptibility of the disease to those therapies. In recent years, several biomarkers of cardiopulmonary damage and inflammatory activity have been studied in animals with *D. immitis*, showing promising results. Pathological levels of D-dimer, a fibrin degradation product, have been reported in dogs with heartworm disease and has been demonstrated that the use of this biomarker provides support for the diagnosis of pulmonary thromboembolism in these animals. Furthermore, concentrations increase according to the severity of the infection and during the adulticide treatment, suggesting the utility of D-dimer measurement to stage and monitor the treated dogs. Regarding cardiac damage, several biomarkers have been evaluated in dogs with heartworm: increases in cardiac troponin I, a very sensitive biomarker of myocardial injury, indicate presence of cardiac insult in infected dogs. This was also demonstrated by the presence of increased circulating levels of myoglobin, another biomarker of cardiac damage. Both biomarkers provide presence, quantify and can be used to monitor myocardial injury in these dogs. Furthermore, NT-proBNP is a natriuretic peptide produced in response to chronic pressure or volume overload, which has shown a correlation between levels of this biomarker and the severity of the disease. Inflammatory processes are also present in dogs infected with *D. immitis*, displayed by the presence of an acute phase response indicated by variations of negative and positive acute phase proteins (APPs). A significant increase in positive APP C-reactive protein (CRP) and decrease in negative APPs albumin and paraoxonase-1 has been observed in infected dogs. It is known that endothelial dysfunction favors inflammatory processes and a relationship between inflammation and hypertension; in heartworm, CRP increases according to the severity, and a strong correlation between pulmonary hypertension and APPs has been observed. In cats, so far only a study in *D. immitis*-seropositive felines reported significantly higher concentrations in some positive APPs, and additional studies are necessary to evaluate if cardiopulmonary or inflammatory biomarkers are useful to assess the diagnosis, severity and monitoring of the disease in this species.

### I12. Where do we go from here? Future priorities in research and practice

#### Genchi C.^1^, Kramer L. H.^2^

##### ^1^Department of Veterinary Medicine, University of Milan, Milan, Italy; ^2^Department of Veterinary Sciences, University of Parma, Parma 43126, Italy

###### **Correspondence:** Genchi C. (claudio.genchi@unimi.it)

The objectives of the European Society of Dirofilariosis and Angiostrongylosis (ESDA) are to: further scientific progress in the study of heartworm and subcutaneous *Dirofilaria* and *Angiostrongylus* infections in Europe; inform the membership and medical and veterinary practitioners of new developments; harmonize procedures for the diagnosis, prevention and treatment of *Dirofilaria* and *Angiostrongylus* infections throughout Europe by writing official guidelines; and to inform animal owners and inhabitants of the importance of these worms and of their prevention. ESDA is committed to the identification of future priorities in research and practice as a necessary step in assuring the continuous development of knowledge and tools that can aid in the management of these diseases. The presence and spread of infection with *Dirofilaria* spp. and *A. vasorum* in Europe have been confirmed. While this is likely due to increased awareness of the parasites by practitioners and physicians, model prediction maps are also of major importance in establishing the risk of these parasites establishing an any geographical area. It is essential to continue studying the factors that favour parasite transmission, growth and survival in both definitive and intermediate hosts (mosquitos, snails, etc.) in order to develop reliable predictive models. While the pathogenesis and clinical presentation of canine heartworm disease is well known, there are still many questions surrounding the cause of clinical signs in heartworm-infected cats and in dogs with *A. vasorum*, in particular the effects on coagulation. Only with further study on how infection causes illness, will it be possible to develop guidelines on how to treat disease in these cases. Perhaps one of the most important research priorities is the development of a reliable diagnostic test for *D. repens* infection in the animal host. The current dramatic spread of this zoonotic parasite is due especially to the lack of clinical signs and therefore the lack of intervention on the part of veterinary practitioners. The availability of a dependable screening tool would not only allow us to monitor the presence and spread of the parasite but would also allow infected animals to be treated quickly, thus breaking the transmission cycle to both other animals and humans. Several drugs are currently available for the treatment and prevention of all three parasitic infections. However, problems with availability, consent, cost and possible resistance have driven research towards the identification of alternative approaches (“integrated approach to prevention”, “alternative adulticide protocols”) which, while promising, require long term follow up studies and further study aimed at identifying the best and most effective (doxycycline vs. minocycline? macrocyclic lactones vs. benzomidazoles?) in the field.

## Oral Presentations

### O1. Survey of canine and human cases of dirofilariosis in parts of Serbia

#### Otašević S.^1^, Momčilović S.^1^, Savić S.^2^, Gajić B.^3^, Gabrielli S.^4^

##### ^1^Department of Microbiology and Immunology, Faculty of Medicine, University of Niš, Serbia, Blvd Zorana Djindjica 81, 18000 Niš, Serbia; ^2^Scientific Veterinary Institute Novi Sad, Serbia, Rumenački put 20, 21000 Novi Sad, Serbia; ^3^Department of Parasitology, Faculty of Veterinary Medicine, University of Belgrade, Serbia, Blvd Oslobodjenja 18, 11000 Belgrade, Serbia; ^4^Department of Public Health and Infectious Diseases, “Sapienza” University of Rome, Piazza le Aldo Moro 5, 00185 Rome, Italy

###### **Correspondence:** Otašević S. (otasevicsuzana@gmail.com)

In last ten years, based on published data, the number of patients with Dirofilaria infection has increased in Southern Serbia [1,2]. However, little is known about canine dirofilariosis in this region of the country. The present study provides an update of *Dirofilaria immitis* and *D. repens* infection status in dogs from Nišava district (Southeastern Serbia). Additionally, aim of this paper is to report and describe two new autochtonous cases of humans, one subcutaneous and second submucosal dirofilariosis caused by *D. repens*. Peripheral blood from 66 outdoors asymptomatic dogs (36 males and 30 females, aged 1 to 14 years) were submited to DNA extraction using commercial kit and molecular amplification of the cytochrome c oxidase subunit I (COI) gene [3], followed by sequencing. Sequences analysis evidenced dirofilariosis in 2 (3%) dogs (one case of *D. repens* and another of *D. immitis* infection). During 2017, two new cases of *D. repens* infection in human patients were diagnosed. The first case was 35 year-old male patient, inhabitant of city of Niš, Nišava district in Southeasten Serbia, who was admitted to the local hospital because of subcutaneous nodule in the antebrachial region. Based on anamnestic data, this nodule persisted for two years. After the clinical examination, patient was reffered to a surgeon with suspicion of lipoma. During the surgical intervention, a complete alive nematode was removed from a nodule and worm was morphologically identified as *D. repens*. The second case was very unusual submucosal human *Dirofilaria* infection which was localised in buccal mucosa and folowed by atopic reaction. The infection has begun with a pronounced edema of buccal mucosa leading to asymmetrical deformity of the face. This hyperactivity disappeared in the next period and a 20 x 15 mm nodule was formed along the lateral edges of maxilla. The nodule was surgically removed and histopathologically analyzed. Microscopic examination of the haematoxylin and eosin-stained preparations demonstrated transverse sections of a helminth which disclosed thick multilayered cuticle with longitudinal ridges (indentations) on the circumference. Subsequent molecular analysis of the remaining tissue sections confirmed the presence of *D. repens* DNA in the sample. New diagnosed cases of human dirofilariosis in the previous year as well as confirmation of *D. repens* and *D. immitis* infection in dogs in areas where no infection has been identified suggest the need for wider research in Central and South part of Serbia.

Written patient consent was obtained for publication.

This study was supported by the Serbian Ministry of Education and Science grant No 175034 and grant No III 41007.

References

1. Tasić-Otašević SA, Trenkić Božinović MS, Gabrielli SV, Genchi C. Canine and human *Dirofilaria* infections in the Balkan Peninsula. Vet Parasitol. 2015;209:151–6.

2. Krstić M, Gabrielli S, Ignjatović M, Savić S, Cancrini G, Ranđelović G, et al. An appraisal of canine and human cases reveals an endemic status of dirofilariosis in parts of Serbia. Mol Cell Probes. 2017;31:37–41.

3. Casiraghi M, Anderson TJ, Bandi C, Bazzocchi C, Genchi C. A phylogenetic analysis of filarial nematodes: comparison with the phylogeny of *Wolbachia* endosymbionts. Parasitology. 2001;122:93–103.

### O2. First molecular investigations on canine dirofilariosis from FYR of Macedonia: preliminary results

#### Gabrielli S.^1^, Giacomi A.^1^, Momčilović S.^2^, Pavlova M. J.^3^, Stefanovski J.^4^, Cancrini G.^1^, Otašević S.^2^

##### ^1^Department of Public Health and Infectious Diseases, Sapienza University of Rome, 00185, Rome, Italy; ^2^Department of Microbiology and Immunology, Faculty of Medicine, University of Niš, Serbia, 18000 Niš, Serbia; ^3^Institute for Microbiology and Parasitology, Medical Faculty, University "Ss Cyril & Methodius", University of Skopje, FY Republic of Macedonia; ^4^Department of Parasitology and Parasitic Diseases, Faculty of veterinary Medicine "Ss. Cyril and Methodius", University of Skopje, FY Republic of Macedonia

###### **Correspondence:** Gabrielli S. (simona.gabrielli@uniroma1.it)

In the last decades, several factors including climate changes, the introduction of new competent vectors, and the movement of infected microfilaremic dogs, have allowed the spread of *Dirofilaria* species towards the northern and north-eastern regions of Europe. A recent review on dirofilarioses in the Balkan Peninsula reported in dogs prevalence from 2 to 43% and from 0.2 to 100% for *D. repens* and *D. immitis*, respectively, and more than 100 cases of human infections due to *D. repens* and 4 to *D. immitis* from 2000 to 2016 [1-2]. Most of recent and extensive studies in the Balkan Peninsula have been carried out in Romania, Serbia, and along the Mediterranean coast of Albania [3]. Notwithstanding Former Yugoslav Republic of Macedonia (FYROM) shows epidemiological features for onset of dirofilariosis, data on *Dirofilaria* infections from this country are scant, not easily found in the international literature and performed using traditional techniques [4-5-6-7]. This study aimed to report preliminary results of the first molecular investigations on canine dirofilariosis from FYROM. Blood samples were collected from dogs enrolled from FYROM (41°8’– 42°08’N; 20°48’– 22°30’E) and adsorbed on filter papers (Whatman grade no.3). Genomic DNA was extracted from dried blood spots and submitted to PCR amplifications of the cox1 gene fragment [8]. A total of 80 dogs were enrolled (48 males, 32 females, mean age 4.95 ± 3.26 years). Out of the total number of included owned dogs, 12 were symptomatic and 68 asymptomatic dogs. PCR amplification yielded amplicons of expected size (about 650 bp) in 7/80 dogs (=8.8%), mostly symptomatics, which were further confirmed by sequencing. Sequence analyses evidenced 100% of identity with *D. immitis* (GenBank: KT716014), for all the specimens. This study reported for the first time a molecular screening for *Dirofilaria* conducted in FYROM, a region of the Balkan Peninsula where only few studies have been reported until now using traditional tools. An overall prevalence of about 8.8% for *D. immitis* was evidenced, suggesting the risk of heartworm infections in the study area. Recently human infections by *D. immitis* were observed and confirmed by molecular methods also in Europe, suggesting that this species, although less frequently, is of zoonotic concern not only in the USA [3]. Further and more extensive surveys as well as entomological samplings are needed to assess the actual prevalence of dirofilariosis in FYROM and to evaluate the vectors of *Dirofilaria* spp. in this country.

References

1. Tasić-Otašević SA, Trenkić Božinović MS, Gabrielli SV, Genchi C. Canine and human *Dirofilaria* infections in the Balkan Peninsula. Vet. Parasitol. 2015;209:151-6.

2. Krstić M, Gabrielli S, Ignjatović M, Savić S, Cancrini G, Ranđelović G, et al. An appraisal of canine and human cases reveals an endemic status of dirofilariosis in parts of Serbia. Mol Cell Probes. 2017;31:37-41.

3. Genchi C, Kramer L. Subcutaneous dirofilariosis (*Dirofilaria repens*): an infection spreading throughout the old world. Parasit Vectors. 2017;10(Suppl. 2):517.

4. Jězic J, Simić C. Prilog poznavanju parazitarne invazije pasa u varoši Skoplju. Jugoslov. Vet Glasnik. 1929;9:383-4

5. Kocevski Z, Atanasovska E, Nikolovski G, Stefanovska J. Presence of *Dirofilaria repens* in professional dogs in the region of Skopje diagnosed with the modified Knott technique. Days of Veterinary Medicine. Ohrid, R. Macedonia, 28–30 October 2010, Book of Abstracts. p. 63.

6. Jurhar-Pavlova M, Cvetković D. Duma H, Kochevski Z, Jankoska G, Petrovska M. Dirofilariasis in R. Macedonia, an overview of literature data and our experience. 46th Days of Preventive Medicine, International Congress. Nis, Serbia, 25–28 September 2012, Book of Abstracts. 130–132.

7. Cirović D, Penezić A, Pavlović I, Kulišić Z, Cosić N, Burazerović J, Maletić V. First records of *Dirofilaria repens* in wild canids from the region of Central Balkan. Acta Vet Hung. 2014;62:481-8.

8. Casiraghi M, Anderson TJC, Bandi C, Bazzocchi C, Genchi C. A phylogenetic analysis of filarial nematodes: comparison with the phylogeny of *Wolbachia* endosymbionts. Parasitology. 2001;122:93-103.

### O3. Investigation on *D. repens* infection in dog shelters in Poland

#### Klockiewicz M.^1^, Dobrzyński A.^2^, Wysmołek M. E.^1^, Nowakowska J.^3^, Długosz E.^1^

##### ^1^Division of Parasitology and Invasiology, Faculty of Veterinary Medicine, Warsaw University of Life Sciences-SGGW, Ciszewskiego Str. 8, 02-786 Warsaw, Poland; ^2^Department of Small Animal Diseases with Clinic, FVM, WULS – SGGW; ^3^Bayer Animal Health, Warsaw, Poland

###### **Correspondence:** Klockiewicz M. (maciej_klockiewicz@sggw.pl)

The skin dirofilariosis of dogs has become a real epidemiological threat in recent years in Poland. It is considered as particularly dangerous for animals staying in shelters or kennels. Thus, an algorithm was established to enhance the treatment and control measures of the infection. The aim of the study was to test the algorithm in different shelter or kennel conditions. The total number of 161 animals (158 dogs and 3 cats) were analyzed. These animals stayed in 8 separate localizations, where episodes of dirofilariosis had previously occurred. As planned only animals older than 1 year (probably exposed to the infection), were included to the study. Animals examined have been previously voluntary reported by owners to diagnose their health status and resolve the problem. Depending on the possibilities following laboratory tests were applied to diagnose the infection: blood smear, differential PCR and ELISA. There were different numbers /ratios/ of infected animals found in particular shelters. These were as follows: 1^st^ – 4 out of 18 (22%); 2^nd^ – 2 out of 17 (12%); 3^rd^ – 11 out of 14 (79%); 4^th^ – 0 out of 14 (0%); 5^th^ – 7 out of 50 (14%) and additionally 1 cat out of 3 (33%) living together; 6^th^ – 4 out of 50 (8%); 7^th^ – 2 out of 93 (2%). Tests in 8^th^ localization were performed twice. The first attempt (using only blood smear method) revealed infection in 28 out of 87 animals (28%), but the second test performed 2 years latter (using all methods) revealed 40 cases out of 74 examined dogs (54%). So far, the general approach of prevention of skin dirofilariosis transmission in dogs depended only on a treatment of individuals which were found positive with blood examination or by surgery. The application of newly established algorithm (including 2 additional methods - differential PCR and ELISA) allowed for more accurate identification of all infected animals in particular localizations. Subsequently, according to the algorithm, the targeted treatment with moxidectin (at recommended doses) was applied to eliminate the infection in all positive animals in particular shelters or kennels. Although these investigations revealed the suitability of the algorithm it is however necessary to continue such approach to decrease the risk of transmission of this potentially zoonotic infection.

### O4. Survey of Italian veterinary practioners on *D. immitis* and *D. repens*.

#### Genchi M.^1^, Rinaldi L.^2^, Cringoli G.^2^, Venco L.^3^, Kramer L. H.^1^

##### ^1^Department of Veterinary Science, University of Parma, Italy, 43121; ^2^Department of Veterinary Medicine and Animal Production, University of Naples Federico II, Italy, 80137; ^3^Clinica Veterinaria Lago Maggiore, Italy, 28040

###### **Correspondence:** Genchi M. (marco.genchi@unipr.it)

Dirofilarial infections (*D. immitis, D. repens*) are vector-borne parasitic diseases mainly of dogs and cats. Moreover, they are zoonotic and endemic areas of both are expanding. The experience of veterinarians is very important for correct prevention and diagnosis. To evaluate this, an electronic questionnaire was sent to all Italian veterinary facilities. In the first months of 2018 a questionnaire was sent by e-mail to 2795 veterinary facilities (surgeries, clinics, hospitals and public facilities). The 31 questions were mainly about in which province the facilities were located, and about diagnosis, prevention and treatment for *D. immitis* and *D. repens* in dogs and cats. In addition, it was asked if they knew the Dirofilariosis societies and if they had attended lectures and/or congresses on the topic. Among 662 responses (27%), 33.7% facilities reported infections only of *D. immitis*, 3.2% only of *D. repens*, 10.7% mixed infections and 52.4% no cases of either parasite in the last year. *Dirofilaria immitis* infections were observed above all in the northern and central Italy. However, also many regions of the south and the islands (Sicily and Sardinia) reported heartworm infections. *Dirofilaria repens* is fairly evenly distributed throughout Italy mainly in co-infections with *D. immitis*. The most frequent diagnostic method used in dogs was the antigen test, 24.0%, followed by the fresh blood smear together with the antigen test 23.3%, aid of a diagnostic laboratory 8.3%, and Knott test together with the antigen test 4.7%. For *D. repens* in dogs results were: diagnostic laboratory 33.8%, skin biopsy 10.8% and fresh blood smear 9.8%. The most frequently diagnostic technique for *D. immitis* in cats was: diagnostic laboratory 27.3%, serological test 26.9% and fresh blood smear 8.2%. For treatment of canine heartworm infection, more then 25% used ivermectin + doxycycline or melarsomin and ivermectin + doxycycline 11%; while more than 50% did not do any treatment in the cat. Prevention was started in the dog in April-May, 54.8%, while 10.3% treated for all year. Finally, more than 70% knew the American Heartworm Society (AHS), while 69% knew the European Society of Dirofilariosis and Angiostrongylosis (ESDA). Our data show how *D. immitis* and *D. repens* are distributed in most of the Italian provinces. Furthermore, the diagnosis is often underestimated and mainly relegated just to serology. This type of research can be a good starting point for scientific societies and to get updated risk maps.

### O5. *Dirofilaria immitis* and the fibrinolytic system. The pivotal role of plasmin in the survival and pathological mechanisms of cardiopulmonary dirofilariosis

#### González-Miguel J.^1^, Diosdado A.^2^, Morchón R.^2^, Simón F.^2^

##### ^1^Institute of Natural Resources and Agrobiology of Salamanca (IRNASA-CSIC), 37008, Salamanca, Spain; ^2^Laboratory of Parasitology, Faculty of Pharmacy, University of Salamanca, 37007, Salamanca, Spain

###### **Correspondence:** González-Miguel J. (javier.gonzalez@irnasa.csic.es)

One of the most striking characteristics of parasites is the adaptation to their hosts for their own benefit. An example of this type of manipulation is the interaction of many different pathogens with the fibrinolytic system of their hosts, which comprises one of the main anticlotting mechanisms of hemostasis. Plasmin is the final enzyme of the fibrinolytic system and displays high, broad-spectrum proteolytic activity by degrading both the fibrin of clots and different components of the extracellular matrix and connective tissue. Accordingly, the recruitment of this enzyme by any blood and/or tissue pathogen could not only be an effective mechanism to avoid possible immobilization by the network of fibrin clots, but also aid the pathogen in its dissemination and stabilization in the host by means of degradation of the different components of the extracellular matrix. However, the large number of substrates on which the proteolytic components of the fibrinolytic system can perform their functions has allowed to relate its over-activation to pathological situations linked with proliferation and migration of vascular cells. Due to its biological characteristics, *D. immitis* supposes a paradigmatic example of this type of adaptations, since it is capable of surviving for long periods of time in the circulatory system of immunocompetent reservoirs, while it produces a vascular chronic parasitosis named cardiopulmonary dirofilariosis. A series of published works have demonstrated that *D. immitis* activates the fibrinolytic system displacing the fibrinolytic balance towards the generation of plasmin. This would imply a survival mechanism by which *D. immitis* could control the formation of clots in its immediate intravascular habitat. However, later works have linked the parasite-dependent over-production of plasmin with the long-term development of the pathogenic mechanisms that occur during cardiopulmonary dirofilariosis. These data demonstrate the dual role of plasmin in dirofilariosis: on the one hand, plasmin could favor the survival of both *D. immitis* and the host, while on the other, it could have a pathological effect at the vascular level. Unfortunately, the key factors on which the predominance of the benefits or drawbacks of this interaction depend are still unknown. A deeper understanding of the way in which *D. immitis* use the fibrinolytic system could result in the definition of important mechanisms in the field of pathogenesis and virulence of parasites, potentially giving new insights into the development of drugs that could be applied not only to dirofilariosis, but also to other plasmin-induced pathologies.

### O6. Human *Dirofilaria repens* infection with pulmonary localization

#### Kucsera I.^1^, Vágó T.^2^, Eltigani A. M.^3^, Fraknói R.^4^, Füstös L.^5^, Kovács K.^5^, Pap-Szekeres J.^5^, Mészáros E.^6^

##### ^1^National Public Health Institute, Department of Parasitology, 1097 Budapest, Hungary; ^2^Bács-Kiskun County Teaching Hospital, Department of Pathology, Kecskemét, Hungary; ^3^Bács-Kiskun County Teaching Hospital, Department of Radiology, Kalocsa, Hungary; ^4^St. Rókus Hospital, Department of Radiology, Baja, Hungary; ^5^Bács-Kiskun County Teaching Hospital, Department of General Surgery, Kecskemét, Hungary; ^6^St. Rókus Hospital, Out Patients’Department of Infectious Diseases, Baja, Hungary

###### **Correspondence:** Kucsera I. (kucsera.istvan@oki.antsz.hu)

Dirofilaria infections are vector-borne parasitic infections mainly of dogs and cats. In Europe, they are caused by *Dirofilaria immitis* and *D. repens*. *Dirofilaria immitis* causes heartworm disease in dogs and cats, while *D. repens* is found mainly in subcutaneous tissues. The intermediate hosts and vectors are mosquitoes of the family Culicidae. Dirofilariosis is an endemic zoonosis in the Mediterranean area and it seems to be an emerging disease in Central Europe. Humans become infected with these worms through mosquito bites. *Dirofilaria immitis* causes pulmonary disease in humans. *Dirofilaria repens* infection in humans manifests as a subcutaneous or ocular nodule, but there were several reports of presentation in unusual body sites, like pulmonary, hepatic, orbital, spermatic cord, abdominal cavity etc. In this report we describe a case of pulmonary dirofilariosis in a patient. The patient had regular pulmonary X-ray screening in July 2014. A suspect nodule was seen in the lower lobe of the left lung, which corresponded to single nodular shadow measuring 10-15 mm on the anteroposterior chest X-ray. The other parts of the lungs were clear. The contrast enhanced chest CT examination showed a well circumscribed homogeneous 18 x 8 mm size mass in 4^th^ segment of the left lung. The lung was emphysematous, without other pathological densities. No pleural effusion or enlarged mediastinal lymph-nodes were seen. In September, the surgical removal of the left lung lesion was performed by VATS technique. The patient was monitored at the Division for Subcritical Care postoperatively, and as their condition become stable, they were emitted from hospital. Examination of the histopathological sections of the nodule revealed cross sectional aspects of helminth. *Dirofilaria repens* was identified on the basis of the morphological characteristics in the histopathological sections. In conclusion: *D. repens* dirofilariosis may have unusual presentation, including pulmonary localization which can be easily confused with benign/malignant tumors in the lung, resulting in surgical intervention. With this presentation the authors would like to call the attention to this possibility.

### O7. Current situation of animal and human dirofilariosis in eastern Romania and Moldova

#### Ciuca L.^1^, Simón F.^2^, Morchón R.^2^, Kramer L.^3^, Genchi M.^3^, Acatrinei D.^4^, Miron L.^4^, Cringoli G.^1^, Rinaldi L.^1^

##### ^1^Department of Veterinary Medicine and Animal Production, University of Naples Federico II, Italy, 80137; ^2^Group of Animal and Human dirofilariosis, Parasitology area, Faculty of Pharmacy, University of Salamanca, Spain, 37007; ^3^Department of Veterinary Medicine, University of Parma, Italy, 43126; ^4^University of Agricultural Sciences and Veterinary Medicine, Iasi, Romania, 700489

###### **Correspondence:** Ciuca L. (lavinia_vet1@yahoo.com)

There is very little information regarding the prevalence of dirofilariosis in dogs and humans in Moldova and Romania, two countries bordered by regions with high incidence of human dirofilariosis. Therefore, the present study was aimed to provide data regarding the prevalence and clinical relevance of dirofilariosis in dogs and humans in some areas from Romania and Moldova. Blood samples were collected from dogs (566) hosted in shelters from 8 counties of Romania and tested with serological and molecular methods for the presence of *D. immitis* and *D. repens*. Moreover, Geographical Information Systems (GIS) were used to produce distribution maps of *D. immitis* and predictive maps based on temperature suitability. Human serum samples (450) collected from Romania and Moldova were analyzed for detection of IgG antibodies against adult somatic antigens of *D. immitis* and *D. repens*. In addition, clinical signs of a patient with ocular dirofilariosis from southern Romania were described. The present study showed a prevalence of *D. immitis* in dogs between 7 and 12% with a high value (60%) in southeastern Romania, instead the prevalence of *D. repens* was higher (15.7%) in the northeastern part of the country. The seroprevalence of human dirofilariosis was 6.9% in Romania and 13.6% in Moldova. Of the 187 individuals from Romania, 13 (6.9%) were positive for anti-*D. immitis* IgG, while 1 patient reacted against both antigens of *D. immitis* and *D. repens*. Of the 263 individuals from Moldova, 36 (13.6%) were positive for anti-*D. immitis* IgG while three (1.4%) recognized both antigens. Only one patient was found positive for anti-*D. repens* IgG. *Dirofilaria repens* worm extracted from the patient was a non-fertile female, containing only oocytes and no developing stages. Considering the main reservoir is represented by the microfilaremic dogs, regular parasitological surveillance and of monitoring dogs are needed in order to define the risk areas of *Dirofilaria* infection in Romania and Moldova.

### O8. Prevalence and diagnostic methods for dirofilariosis in dog population from azylum and shelters in Vojvodina, Serbia

#### Savić S.^1^, Gabrielli S.^2^, Žekić M.^1^, Marčić D.^1^, Gajić B.^3^, Momčilović S.^4^, Potkonjak A.^5^, Ćurčin L.^6^, Otašević S.^4^

##### ^1^Scientific veterinary institute “Novi Sad”, Novi Sad, Serbia, 21000 Novi Sad; ^2^Department of Public Health and Infectious Diseases, “Sapienza” University of Rome, 00185 Rome, Italy; ^3^Department of Parasitology, Faculty of Veterinary Medicine, University of Belgrade, Serbia, 11000 Belgrade, Serbia; ^4^Department of Microbiology and Immunology, Faculty of Medicine, University of Niš, Serbia, 18000 Niš, Serbia; ^5^Department for veterinary medicine, Faculty of Agriculture, University of Novi Sad, Novi Sad, Serbia; ^6^Veterinary practice “Intervet“, Belgrade, Serbia

###### **Correspondence:** Savić S. (sara@niv.ns.ac.rs)

Canine dirofilariosis is a vector borne disease, spread by the mosquitoes. During the last 20 years, dirofilariosis has spread over the majority of Serbia, but the highest number of cases can be found in Vojvodina. Climate and air humidity in this part of the country is pretty much in favour of mosquito life-cycle and therefore also in favour of *Dirofilaria* spp. development within the mosquitoes. Stray dogs which come to a shelter or azylum are usualy considered to be without any preventive protection against any vector-borne disease. During the period 2015-2017, a total number of 482 dog blood samples was taken from dogs entering the shelters and aziles, which were then analysed for the presence of microfilaria by Knot test and adult forms of *D. immitis* by ELISA test (VetLine *Dirofilaria* Antigen, Nova Tec). From 482 samples, 5.4% were found positive for the presence of microfilariae and 8% were positive for the presence of adult forms antigen (Ag). Out of the 482 samples, 324 (4.3% positive for microfilariae and 3.1% of samples positive for the adults Ag) were analysed also by molecular methods (DNA extraction and molecular amplification of the cytochrome c oxidase subunit I (COI) gene, followed by sequencing and compared with the other two methods. All of the samples positive for microfilariae were also positive by PCR. Several samples were positive only by modified Knot test 2.47% (8). Some of the samples were positive only by ELISA 1.54% (5) which could indicate an occult infection. Using PCR method, out of 18 samples that were found positive only one was identified as *D. repens* and all the others were found to be *D. immitis*, suggesting that the occurence of *D. immitis* is more often in Vojvodina region than *D. repens*. One sample identified as PCR-positive for *D. repens* was neither positive by ELISA nor Knot test. From the total of 17 PCR positive samples for *D. immitis*, 9 were also positive by Knot test (52,9%), 3 by ELISA (17.6%) and 5 by both mentioned tests (29.4%). It can be concluded that the prevalence among stray dogs in Vojvodina region is somewhere between 5 and 8%, that more prevalent is *D. immitis* than *D. repens*. Additionally, applaying of only one diagnostic method for microfilaria of adults Ag detection for diagnostics, could cause that some of the positive cases will be missed.

### O9. Synergism of ML/doxycycline adulticide effect: an in vitro model

#### Lucchetti C.^1^, Genchi M.^1^, Venco L.^2^, Balogh L.^3^, Kramer L.^1^

##### ^1^Faculty of Veterinary Medicine, University of Parma, Italy, 43126; ^2^Veterinary Hospital “Città di Pavia”, Italy, 27100; ^3^Ministry of Human Capacities (EMMI), Hungary, H-1221

###### **Correspondence:** Lucchetti C. (chiara.lucchetti1@studenti.unipr.it)

Due to its obligate nature in filarial parasites, the bacterial endosymbiont *Wolbachia* have been a target for several drug discovery initiatives. Recent studies have shown that antibiotic treatment of *Dirofilaria immitis*-infected dogs can inhibit parasite embryogenesis, larval development, microfilarial production, and long-term survival of adults [1]. Recent studies have shown that administration of doxycycline (DOXY) in combination with the macrocyclic lactone ivermectin (IVM), a well-known anthelmintic drug, used both for HWD prevention as well as for its slow-kill effects on adult parasites, provided a more rapid and stronger adulticidal and microfilaricidal effect, than either of the two alone [1, 2]. Although, previous experiments have shown that ABC transporter, proteins acting as efflux pumps for various drug, are inhibited by IVM [3]; the actual mechanism laying behind the increase in efficacy of the combination therapy is still unkown. This study proposes to develop an in vitro model, using adult parasites *D. immitis*, to study the effects of different drug treatment regimens on the ABC transporter activity. Adult worms *D. immitis* were collected from naturally infected dogs. They were treated with different combinations of the two drugs. RNA was extracted from each treated parasite and cDNA prepared for expression study of ABC transporters. The first steps of the methodology optimization were carried out and a collection of adults of *D. immitis* treated with/without DOXY alone, IVM alone or a combination of the two, for both 24 and 48 hours, was obtained. Moreover, per each of the treated worms, pure RNA was extracted and cDNA prepared. The results of the RT-PCR will be presented. In this study, we were able to optimize the first steps necessary for the development of an in vitro assay aimed at elucidating the molecular mechanisms laying behind the marked filaricide effects of a combination of MLs and DOXY against *D. immitis*, this by focussing on the effects of these molecules on ABC transporters activity.

References

1. Kramer LH, Genchi C. Where are we with Wolbachia and doxycycline: an in-depth review of the current state of our knowledge. Vet Parasitol. 2014;206:1-4.

2. McCall JW, Genchi C, Kramer LH, Guerrero J, Venco L, et al. Heartworm disease in animals and humans. Adv Parasitol. 2008;66:193-285.

3. Ballent M, Lifschitz A, Virkel G, Sallovitz J, Maté L, Lanusse C. In vivo and ex vivo assessment of the interaction between ivermectin and danofloxacin in sheep. Vet J. 2012;192:422-7.

### O10. Heartworm control in Grenada, West Indies - Results of a field study using imidacloprid 10% + moxidectin 2.5% spot-on (Advantage Multi®) and doxycycline for naturally acquired *Dirofilaria immitis* infections

#### Paterson T., Fernandez C., Burnett P., Lessey L., Hockley T.

##### St. George’s University, School of Veterinary Medicine, St. George’s, Grenada, W.I.

###### **Correspondence:** Paterson T. (tpaterson@sgu.edu)

*Dirofilaria immitis* is a filarial parasite of wild and domestic canids around the world. It is most prevalent in tropical and sub-tropical climates where favorable climatic conditions support the parasite’s life-cycle. Treatment with melarsomine dihydrochloride is effective in eliminating adult *D. immitis* worms, however the high risk of post-therapy pulmonary thromboembolism, inconsistent drug availability and expense of treatment warrants exploration of alternative treatment options for canine heartworm disease. The purpose of this randomized controlled field study was to compare the adulticidal efficacy of a combination therapeutic protocol using 10% imidacloprid + 2.5% moxidectin spot-on (Advantage Multi^®^; Bayer, Germany) and a single 28-day course of doxycycline with that of melarsomine dihydrochloride (Immiticide^®^; Merial, Lyon, France). Thirty-seven naturally-infected domestic dogs with heartworm disease were enrolled in the field trial. Thirty dogs were evaluated for a minimum of 12 months. Seven dogs were withdrawn from the study (3 canine ehrlichiosis, 2 non-compliance, 2 wrongful inclusion). Dogs were randomly assigned to either the control (CP)(n=15) or investigational treatment product (IVP) group (n=15). Dogs in the control group received two deep intramuscular injections of Immiticide^®^ (2.5 mg/kg) 24 hours apart (or using a split protocol on days 0, 28 & 29 in the case of early stage 3 disease). Dogs in the investigational treatment group were treated with oral doxycycline (10 mg/kg twice daily x 28 days) and application of Advantage Multi^®^ once monthly for a total of 9 doses. Dogs were evaluated every four weeks for 9 months, then every twelve weeks up to an additional 9 months. Data from the IVP group was analyzed for non-inferiority as defined by a 15% margin of difference. Parasiticidal efficacy was based on antigen status using PetChek^®^ 34 Heartworm-PF Antigen test (IDEXX Laboratories, Maine, USA). Based on the number of dogs with no detectable antigenemia, non-inferiority of the IVP group was confirmed at months 12, 15 and 18. Non-inferiority was also confirmed after heat-treating samples for month 12 and month 18, but was missed for month 15. Non-inferiority was also supported by echocardigraphy at month 12. In conclusion, monthly application of Advantage Multi^®^ when combined with an appropriate course of doxycycline is an effective adulticidal therapy for canine heartworm infection and represents a safe alternative to treatment with melarsomine dihydrochloride-particularly in cases where financial constraint or debilitated health may preclude treatment of an infected individual.

This study was supported by Bayer Animal Health.

### O11. Characterization of the humoral response in dogs infected with *Dirofilaria repens*

#### Długosz E.^1^, Wysmołek M. E.^1^, Dobrzyński A.^1^, Zawistowska-Deniziak A.^2^, Klockiewicz M.^1^

##### ^1^Faculty of Veterinary Medicine, Warsaw University of Life Sciences-SGGW, Poland, 02-777; ^2^Witold Stefański Institute of Parasitology, Polish Academy of Sciences, Poland, 00-818

###### **Correspondence:** Długosz E. (ewa_dlugosz@sggw.pl)

Skin dirofilariosis caused by *Dirofilaria repens* is an emerging zoonotic disease transmitted by mosquitoes. So far there are no reports describing the humoral response in skinworm infected dogs. The aim of the study was to evaluate *D. repens* specific antibody titers. One hundred and fourty nine blood and serum samples were collected within Warsaw agglomeration from dogs suspected for skin dirofilariosis. The infection was confirmed using PCR. Adult worm somatic antigen-specific IgG, IgE and IgM antibodies were titrated using ELISA, moreover IgG1/IgG2 ratio was calculated. The specificity of ELISA was confirmed using Western blotting. Fourty one percent of collected samples were found positive in PCR. IgG titers (log10) in those dogs ranged from 3.2 to 5.3. Interestingly, 50% of PCR negative dogs showed IgG titers higher than 3.2, nonetheless moderately strong relationship between PCR result and IgG titers was noted. An average IgG1/IgG2 ratio was significantly higher in PCR-positive dogs comparing to negative ones. IgE and IgM titers (log10) ranged to 3.1 and 4.7 respectively; however the relationship of these titers with PCR result was relatively weak. To our knowledge this is the first report describing *D. repens* specific antibody titers in infected dogs. Our results suggest that in many cases skin dirofilariosis might be underdiagnosed. This clearly shows that diagnostic methods should be complemented with a serological test to efficiently detect skinworm infections. This study shows that from the three examined antibody classes only specific IgG could be considered as a marker of the infection, as neither IgE or IgM titers were correlated with PCR result. Moreover, IgG1/IgG2 ratio could be perhaps helpful in the prediction of the phase of infection as in microfilaremic PCR-positive dogs higher ratio values were noted. In this study we were not able to discriminate between the IgG response specific to skinworms or to its *Wolbachia* endosymbiont bacteria. More experiments need to be performed in order describe the IgG response in detail what is necessary to characterize the course and the pathology of the infection.

### O12. Minimally invasive surgical heartworm removal as first line therapy in dogs showing HW on echocardiographic examination

#### Peterfia D.^1^, Laczko T.^1^, Szabo I.^1^, Venco L.^2^

##### ^1^Minivet Állatorvosi Rendelő, 1162 Budapest, Hungary; ^2^Clinica veterinaria Lago Maggiore, Italy

###### **Correspondence:** Peterfia D. (dpeterfia25@yahoo.com)

As suggested by the ESDA guidelines, surgical heartworm removal should be the therapy of choice when possible, because this is the only one that can avoid pulmonary thromboembolism following successful pharmacological adulticide therapy. The purpose of this study was to evaluate the efficacy and safety of surgical removal in dogs, regardless their clinical conditions. Inclusion criteria was the visualization of Hws during echocardiography in the pulmonary arteries and the owner’s consent after detailed explanation of advantages and risks. For every dog, weight, age, clinical conditions (symptomatic or asymptomatic) and presence or absence of PHT (based on tricuspidal regurgitation, size of the pulmonary arteries and RPAD Index) was considered together with the total numbers of worms removed. Surgery was performed with Flexible Alligator Forceps following the Ishihara technique as well described in literature. Surgery was considered: Successful (S) if no worms could be visualized by echocardiography soon after surgery. Incompletely Successful (IS) if still some filariid worm echoes could be visualized at the end of the procedure. Unsuccessful (U) if no worms could be removed. Every dog was treated after surgery with Doxycycline 5-10 mg/kg twice a day oral route for 30 days and topical moxidectin (2,5 mg/kg) once a month for 7 months for avoiding development of pre-existing larval stages. After 7 months, dogs were re-evaluated both by echocardiography and antigen testing and considered definitively cured if negative for both. Any worsening of clinical condition or echocardiographic parameters was also considered. Between 2016 and 2017, 37 privately owned dogs (24 females, 13 males) were included into the study and underwent Surgical HW removal. Thirty-one of them were asymptomatic, 6 were symptomatic. Age ranged from 2 years to 10 years (average 4.8 years. Weight ranged from 1.8 kg to 27 kg (average 9.4 kg). Surgery was considered S in 20 dogs (54%), IS in 11 (30%), and U in 6 (16%) dogs. The total worms removed was 146 with an average of 4 in each dog. No fatalities occurred during surgery. After 7 months, 27 (87%) out of 31 dogs in which worms were removed were considered definitively cured yielding negative antigen test result while 4 dogs were still positive on antigen testing and/or echocardiography. All dogs in group S were cured after 7 months (100%) while 7 (64%) dogs in the IS group became negative on antigen testing and echocardiography at the 7 months recheck period. No dogs experienced clinical or echocardiographic worsening at the 7 month recheck. Six out of 6 symptomatic patients had clinical improvement. Six patients had echo improvement. Minimally Invasive Surgical Heartworm Removal was a completely safe (100%) and very effective (87%) therapy in every set of clinical conditions when HWs can be visualized by echocardiography and in these cases, it should be considered the elective therapy.

**Fig. 1 (abstract O12).**
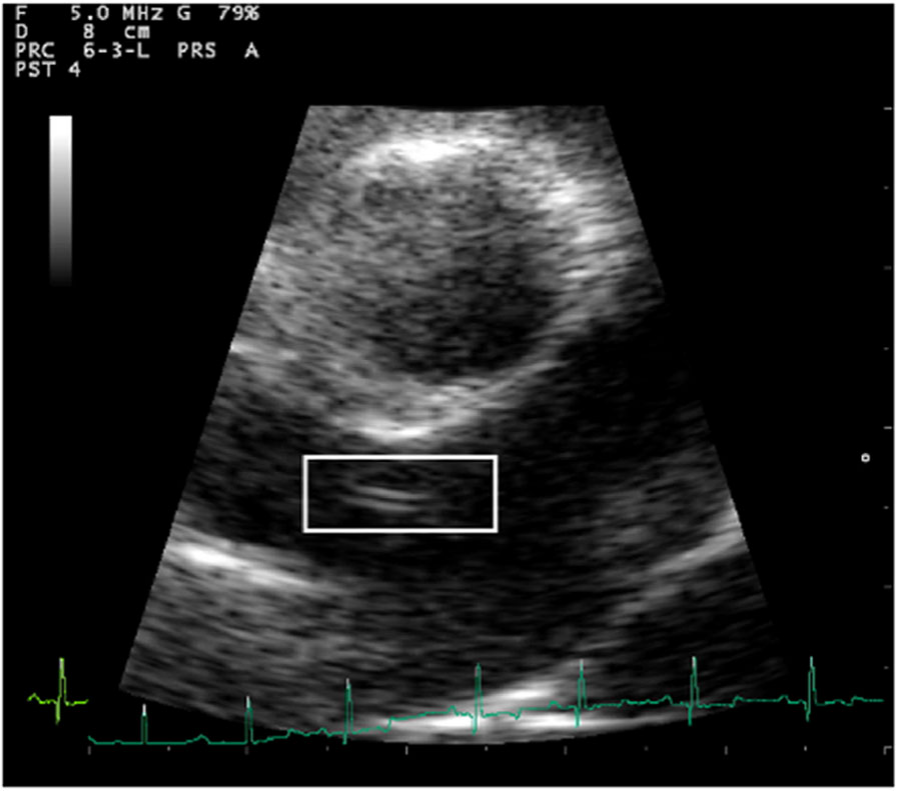
Echocardiography: right parasternal short axis view, showing double lined echoes (square) into the right pulmonary artery suggesting the presence of an adult heartworms

**Fig. 2 (abstract O12).**
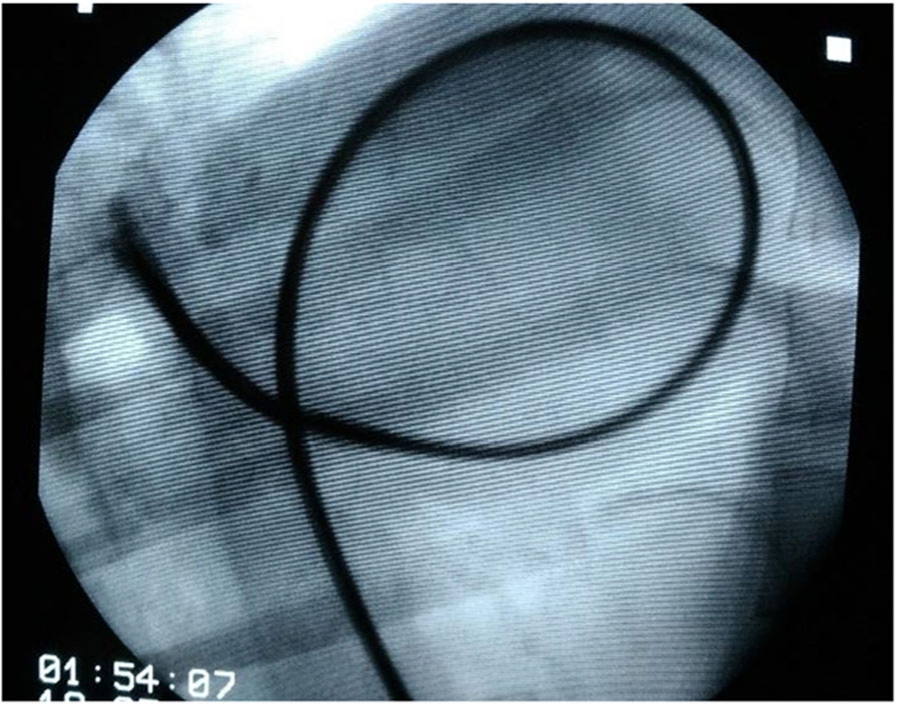
Flexible Alligator Forceps under fluoroscopic guidance is introduced into the right pulmonary artery. The jaws are open suggesting that some heartworms are grabbed

**Fig. 3 (abstract O12).**
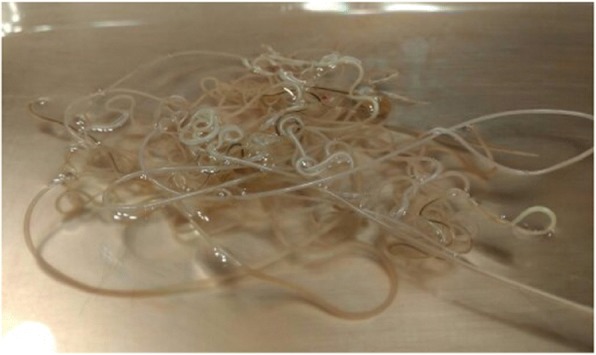
Heartworms removed at the end of a surgery

### O13. Medical treatment of caval syndrome in a dog

#### Kalinov T. (drkalinov80@gmail.com)

##### Zaravet Veterinary Clinic, Bulgaria, 4000

Caval syndrome is the most severe complication in heartworm disease, developing after migration of adult dirofilaries in right ventricle, right atrium and vena cava. The reason for it is usually high worm burden and severe pulmonary hypertension [1]. Because of the cruelty of the condition, immediate removing of the worms with surgical extraction is recommended [2]. In this case we successfully managed caval syndrome in a dog only with medical treatment. According to the author's knowledge, there are no publications of treatment of caval syndrome without surgical extraction. Bulit is a 10 years-old, male, intact, Bulgarian Shepherd dog. He is regularly vaccinated and dewormed. Several months before the diagnosis he has started to lose weight, he has become lethargic and he has exercised intolerance and occasional coughing. The owners thought that these are geriatric signs. Before coming to the clinic, the dog has stopped eating and drinking and could not stay on his legs. We diagnosed class 4 heartworm disease with caval syndrome. Laboratory tests showed leukocytosis, thrombocytopenia, increased ALAT, ALP, BUN. Antigen test for heartworm was positive. Echocardiography revealed dilated right ventricle, right atrium, pulmonary artery and worms attached to the tricuspid valve. Left ventricle and atrial were collapsed and barely visible. There was free fluid in the abdomen. The owners refused surgical extraction, so we decided to use slow kill protocol. We prescribed sildenafil 1mg/kg/bid, doxycycline 10mg/kg/sid for one month and topical moxidectin/imidaclopride (Advocate Bayer). After a weak the dog was better, left ventricle was dilated but with poor fractional shortening. We hypothesized that the dog has primary dilated cardiomyopathy, so we added pimobendane 0.25 mg/kg/bid and benazepril 0.5 mg/kg/bid (Fortecor Plus Elanco), and prednisolone 0.5 mg/kg/sid for prevention of pulmonary thromboembolism. Two weeks later we did not see heartworms in the right heart nor in the pulmonary artery. We stopped doxycycline after one month and continued with the other medications. Six months after the diagnosis, the test for heartworms was negative. The dog is feeling well, has moderate pulmonary hypertension with exercise intolerance, he gains weight and there are no any other signs.

References

1. Strickland KN. Canine and feline caval syndrome. Clin Tech Small Anim Pract. 1998;13:88-95.

2. Bové CM, Gordon SG, Saunders AB, Miller MW, Roland RM, Achen SE, et al. Outcome of minimally invasive surgical treatment of heartworm caval syndrome in dogs: 42 cases (1999-2007). J Am Vet Med Assoc. 2010;236:187-92.

### O14. Pulmonary hypertension persists in dogs with heartworm 6 months after being treated

#### Falcón-Cordón Y.^1^, Falcón-Cordón S.^1^, Montoya-Alonso J. A.^1^, Caro-Vadillo A.^2^, Carretón E.^1^

##### ^1^Internal Medicine, Faculty of Veterinary Medicine, Research Institute of Biomedical and Health Sciences (IUIBS), University of Las Palmas de Gran Canaria, 35411, Spain; ^2^Department of Animal Medicine and Surgery, Veterinary Faculty, Complutense University of Madrid, 28040, Spain

###### **Correspondence:** Carretón E. (elena.carreton@ulpgc.es)

Pulmonary hypertension (PH) in heartworm disease is mainly caused by intimal proliferation of the pulmonary arteries and presence of pulmonary thromboembolisms. To determinate PH in dogs infected by *Dirofilaria immitis*, the Right Pulmonary Artery Distensibility Index (RPAD Index) is an echocardiographic index that has been validated by several studies. A recent research showed that 1 month after the last dose of melasomine dihydrochloride neither significant aggravation nor improvement of the pulmonary damage was observed in the treated dogs and concluded that probably more time was needed before appreciating some positive changes. Therefore, this study evaluated the RPAD Index 6 months after the last adulticide treatment to determine the evolution of the pulmonary pressure and endarteritis after the elimination of the worms. The study included 13 heartworm-infected dogs brought to the Veterinary Medicine Service of the University of Las Palmas de Gran Canaria to receive adulticide treatment following the management protocol recommended by the European Society of Dirofilariosis and Angiostrongylosis. Animals were further evaluated for the presence or absence of microfilariae using a modified Knott test. Physical exam, thoracic radiographies and echocardiography exam were performed at the beginning and at the end of the study. The RPAD Index and the worm burden were echocardiographically assessed following specific guidelines. Mean RPAD Index at the beginning of the adulticide treatment was 29.3±9.9%; PH was present in 56.25% of the dogs (18.75% severe PH, 31.25% moderate PH and 6.25% mild PH). Six months after the last dose of melarsamine, mean RPAD Index was 27.8±9.6%; PH was present in 62.5% of the dogs (18.75% severe PH, 25% moderate PH and 18.75% mild PH). No statistically significant differences were observed between RPAD indices at the beginning and the end of the study. The results through the evaluation of the RPAD Index showed that the elimination of the worms did not appear to have any positive influence over the pulmonary pressure and presence of endarteritis. A previous study showed that no improvement was observed one month after the last dose of melarsomine, concluding that probably parasite remnants and circulating antigens were still present. Although there are evidences that after the adulticidal therapy, intimal proliferation is partially reversible, the results show that PH did not improve 6 months after the elimination of the parasites and the endarteritis seems to persist. This study showed the utility of the RPAD Index in the evaluation of the dog after being treated for heartworm disease.

### O15. First reported case of symptomatic *Dirofilaria immitis* infection in a housoled domestic ferret (*Mustela putorius furo*) in Bulgaria

#### Mihaylova L. (lillyvet@gmail.com)

##### Veterinary surgeon in United Veterinary Clinic Varna (Bulgaria)

Heartworm disease in dogs and cats is well known in many European countries including Bulgaria. There are also studies confirming dirofilariosis in wild foxes and *Canis aureus* but no reports about heartworm disease in domestic ferrets in our country. A 5 year-old male, entire, pet ferret (*Mustela putorius furo*), weight 0.9 kg was presented with labored abdominal breathing. The owner reported reduced appetite, difficulty breathing and restlessness. The ferret was not able to sleep or lie down for more than few minutes. The ferret was used to live mainly indoors and was allowed to be outside in the garden, during the summer for just few hours during the day, to be exposed to natural sunlight. On presentation ferret was lethargic with abdominal breathing and breathing rate up to 90/minute. There was clear subcutaneous edema more prominent on the front and hind legs and ventral part of the abdomen. Mucous membranes were pale, while CRT was not possible to be assessed. Heart rate ranged in between 120-180 bpm. Pulses were weak even if assessing on the femoral artery was difficult due to the subcutaneous edema. Abdominal palpation was unremarkable and lymph nodes were normal in size. Thoracic radiograph showed loss of detail in the thoracic cavity consistent with pleural effusion. Thoracic US was performed confirming pleural effusion and 120 ml of modified transudate was drained. Brief screening echocardiography showed normal left atrium and left ventricle and severely dilated right atrium containing double line hyperechoic objects suggesting the presence of several adult heartworms (Fig 1). Right atrium was larger than left atrium. Doppler study and any further detailed investigation of the heart were not possible to be performed due the fact that the ferret became aggressive and the owner declined any sedation or anesthesia. Snap^®^ HTWM Antigen test (Idexx) on blood was negative and at fresh blood smear examination no microfilariae were possible to be identified. Knott test was not possible to be performed due to limited amount of sampled blood. On the basis of echocardiography findings diagnosis of HW disease was done. Negative HW antigen test was assumed to be due probably due to juvenile *D. immitis* worms and right atrium localization to the small size of pulmonary arteries as described in cats and ferrets. The ferret was treated with Advocate^®^ spot on >4kg (half tube), Furosemide 2mg/kg twice a day and Prednisolone 1mg/kg daily both orally. The ferret was stable on that therapy. He was eating and drinking well regain the normal body weight 1.5 kg. no breathing difficulties were reported. He was rechecked 35 days after initial presentation. Echocardiography showed right mildly dilated atrium but no presence of HW (Fig 2). Only 10 ml of fluid was drained from the thoracic cavity. From that time he was stable with no owners complain for 6 month. Suddenly he developed respiratory distress and on presentation was with cyanotic membrane. Pulmonary thromboembolism connects to HW disease was suspected Owner elected euthanasia and no more investigations. Necropsy was declined. This case shows the in endemic area even indoor domestic ferrets may be infected with *Dirofilaria immitis* and that the disease is difficult to be diagnosed and can lead to death. Suspicion about this problem and monthly chemoprophylaxis should be warranted in this situation as in dogs and cats.

**Fig. 1 (abstract O15).**
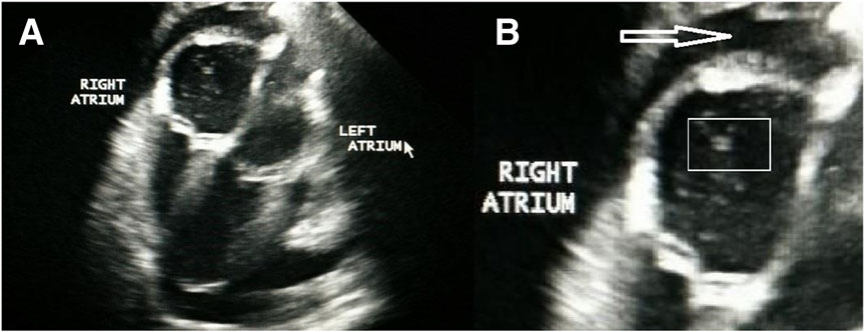
Four chambers apical view, showing enlarged right atrium, double lined echoes (square) into the right atrium suggesting the presence of Heartworms, and small amount of pleural effusion

**Fig. 2 (abstract O15).**
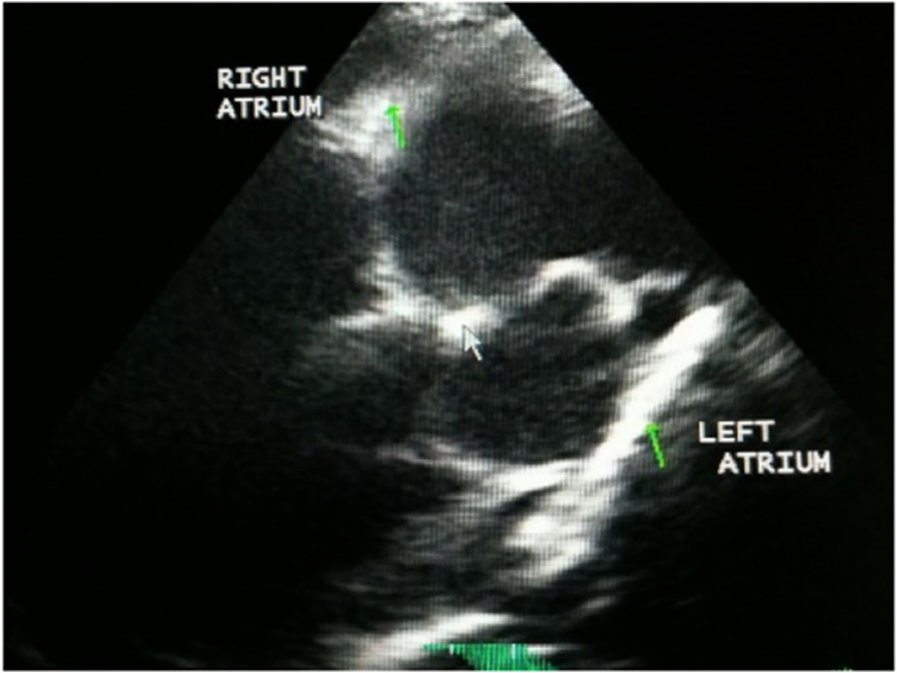
Echocardiography of the same ferret 35 days later, showing mildly dilated right atrium, but no presence of heartworm

### O16. Italian nationwide survey on filariosis and angiostrongilosis

#### Brianti E. (ebrianti@unime.it)

##### Department of Veterinary Sciences, University of Messina, Polo Universitario Annunziata, 98168, Messina, Italy

A sample of 1748 dogs, enrolled by 13 regional units, was examined for *Angiostrongylus vasorum* and filarial infections. Dogs were physically examined, sampled for blood and faeces, and data on treatments collected through a questionnaire. Faeces (5 g) were analyzed for the presence of *A. vasorum* larvae using Baermann method. Blood samples were examined for blood microfilariae by Knott’s test, and the sera analyzed with a rapid serological assay for the detection of *A. vasorum* circulating antigens (Angio Detect^®^). The study population aged 6 months to 17 years (mean 58.2 months), was gender-balanced (50.6% females and 49.4% males), and uniformly distributed along the country, being 31.8% of the enrolled animals from northern, 29.2% from central and 39.1% from southern regions. All the dogs had regular/constant outdoor access. Overall 37 dogs (2.1%) scored positive to *A. vasorum*, the majority of them (25) tested positive to both Baermann and serology, while 8 and 4 animals scored positive only to serology or Baermann, respectively. Infection by *A. vasorum* was patchy distributed with the highest prevalence in central regions (up to 7.14%). Despite the infection was diagnosed in animal of all ages, the frequency was twice (4.4%) in dogs aged 6-12 months compared to other age classes (1.4-2.1%). A few infected animals (29.7%) displayed suggestive signs while the vast majority was apparently asymptomatic. Prevention with products active against respiratory nematodes was not common in examined dogs and only 104 (5.9%) dogs were treated with products with activity against the parasites. Circulating microfilariae were found in 52 (3%) blood samples and identified as *Dirofilaria repens* (1.5%), *Dirofilaria immitis* (1.1%) and *Acanthocheilonema reconditum* (0.8%). The majority of animals (82.7%) was infected by a single filarial species. Nearly half (869) of the examined dogs were living in areas recognized as endemic for *D. immitis*. However, only 42.2% (367) was reported to be treated with anthelmintic effective for heartworm prevention; of this latter group, only 62.4% (229) was treated with appropriate products/regimens, while 23.2% was treated with inadequate regimens, or with off label products (14.4%). Both angiostrongylosis and filarioses are wide-present in Italy, and despite the low prevalence observed especially for filarioses, the insufficient adoption of effective preventative measures may favour the recrudescence.

This study was supported by Boehringer-Ingelheim Italy

### O17. “Regard croisés” on canine dirofilariosis and leishmaniosis. Diagnosis and prevention

#### Alves L.^1^, Carretón E.^2^, Franc M.^3^, Kostadinov M.^4^, Koutinas C^5^, McCall J.^6^, Mirò G.^7^, Oliva G.^8^, Peterfia D.^9^, Petersen C. A.^10^, Pouzet E.^11^, Varloud M.^11^, Venco L.^12^

##### ^1^Federal Rural University of Pernambuco, Recife, Brazil; ^2^University of Las Palmas de Gran Canaria, Spain; ^3^Ecole Nationale Vétérinaire de Toulouse, Toulouse, France; ^4^Veterinarna Klinika Novet, Plovdiv, Bulgaria; ^5^University of Thessaloniki, Thessaloniki, Greece; ^6^University of Georgia, Athens, USA; ^7^Veterinary Faculty, Universidad Complutense of Madrid, Madrid, Spain; ^8^Department of Veterinary Medicine, University of Naples Federico II, Italy; ^9^Equi-Sound Kft, Budapest, Hungary; ^10^University of Iowa, Iowa city, USA; ^11^Ceva Santé Animale, Libourne, France; ^12^Clinica Veterinaria Lago Maggiore, Novara, Italy

###### **Correspondence:** Oliva G. (gaeoliva@unina.it)

Canine dirofilariosis and leishmaniosis are two major vector-borne diseases of increasing importance across the world. Because of their overlapping and spreading distribution, practicing veterinarians may have to manage both diseases. Despite the different pathogenic agents (protozoa/helminth), the two diseases share a number of characteristics. Dirofilariosis and leishmaniosis are transmitted by flying insects, mosquitoes and sandflies, respectively. Both of diseases have long latency/prepatent periods and co-infections with these parasites can occur in endemic areas, hindering the work of clinicians. Clinical signs are variable, diagnosis is complex, prognosis is often guarded, and a safe and effective treatment is currently lacking for leishmaniosis. This cross-review benefits different expertises including epidemiology, veterinary parasitology, entomology and veterinary practice in order to obtain a simple and clear document for practitioners. The topics include etiology, epidemiology, travel and risk factors, clinical presentation, diagnosis, clinical staging and prognosis. A diagnosis and prevention decision-tree were consolidated for routine veterinary practice. The importance of prevention is emphasized. The superior prevention strategy to be presented relies on a recommended, novel multimodal approach known as DOUBLE DEFENSE^TM^ to fight the vectors and the diseases.

### O18. *Dirofilaria immitis* interferes with the coagulation cascade of the host

#### Diosdado A.^1^, García M. C.^2^, Silva S.^2^, Pérez C.^2^, Morchón R.^1^, Simón F.^1^, González-Miguel J.^3^

##### ^1^Laboratory of Parasitology, Faculty of Pharmacy, Animal and human dirofilariosis group, University of Salamanca, Salamanca, Spain, 37007; ^2^Veterinary Clinic La Vega, Salamanca, Spain, 37008; ^3^Laboratory of Parasitology, Institute of Natural Resources and Agrobiology of Salamanca (IRNASA-CSIC), Salamanca, Spain, 37008

###### **Correspondence:** Diosdado A. (alidm@usal.es)

*Dirofilaria immitis* is a parasitic nematode that survives in the circulatory system of immunocompetent reservoirs for many years, causing the canine and feline cardiopulmonary dirofilariosis. One of the most life-threatening events of this disease is the appearance of thromboembolisms, especially when simultaneous death of groups of adult worms occurs, suggesting that the parasite could develop mechanisms that interfere with the formation of blood clots as a survival mechanism. In this sense, the interaction between *D. immitis* and the fibrinolytic system of the host, the main anticoagulant mechanism of haemostatic system in vertebrates, has already been demonstrated. It was shown that excretory/secretory and surface associated antigens from *D. immitis* bind plasminogen and stimulate the generation of plasmin, enzyme responsible for degrading fibrin clots. Fibrin is the final product of the coagulation cascade, a process in which clotting factors trigger a series of enzymatic chain reactions divided into extrinsic and intrinsic pathways, which converge in a common pathway that finishes with the formation of thrombin, the enzyme responsible for converting fibrinogen into fibrin. The present work evaluates the possible interference of *D. immitis* with the coagulation cascade in order to increase the knowledge on the study of the interaction between the parasite and the haemostatic system of the host. The anticoagulant activity of excretory/secretory antigens from *D. immitis* adult worms (DiES) was evaluated in blood samples from healthy dogs. Assays of prothrombin time (PT) and activated partial thromboplastin time (APTT) were respectively performed to evaluate the extrinsic and intrinsic pathways, as well as the common pathway of coagulation. In addition, a thrombin time (TT) assay was performed to evaluate the function of fibrinogen. Blood samples in the presence of equal volumes of PBS were used as negative controls. The values of PT and APTT are modified in the presence of DiES given the optical densities were significantly lower in the presence of the antigens over time (p < 0.05). Conversely, no statistically significant differences were observed in TT assays between groups. *Dirofilaria immitis* interferes with extrinsic and/or intrinsic pathways of the host before transformation of fibrinogen into fibrin since the parasite does not alter the function of fibrinogen. This interference could be used by the parasite as a survival mechanism in order to control the formation of clots in its immediate intravascular habitat.

## Poster Presentations

### P2. Molecular cloning of Dre-33 cDNA and bioinformatic evaluation of its suitability as a diagnostic antigen for cutaneous dirofilariosis

#### Pękacz M., Bąska P., Długosz E., Wysmołek M. E., Klockiewicz M., Wiśniewski M.

##### Faculty of Veterinary Medicine, Warsaw University ofLife Sciences – SGGW, Nowoursynowska 159, 02-776 Warsaw, Poland

###### **Correspondence:** Długosz E. (ewa_dlugosz@sggw.pl)

*Dirofilaria repens* is a causative agent of subcutanoeus dirofilariosis. As a result of climate change and human activities these nematodes have spread into central and eastern parts of Europe. Although dogs are the main hosts these parasites can also infect cats and humans. The aim of the research was to clone so far undefined cDNA encoding Dre-33antigen. Dre-33 is a 33kDa pepsin inhibitor. This protein has been characterized in other filarial worms such as *Onchocerca volvulus*, *Dirofilaria immitis*, *Brugia malayi*, but also in nematodes such as *Toxocara canis*. It has been described as highly immunogenic and potentially useful as a diagnostic antigen. Dre-33 is located in muscle tissue and in female reproductive organs and is secreted by females in the amount of 10 ng per day. Total RNA was isolated from the homogenate of mature worm and reverse transcribed into cDNA using PTX primer (5'-GAA CTA GTC TCG AGT TTT TTT TTT TTT TTT T-3'). In order to amplify 5' and 3' ends of the sequence RACE-PCR technique was applied. 3' RACE - PCR was performed with reverse primer PTX and forward primer Dre33For (5'-GAT GGY TGY ATG GTT CAG AAT-3'). A poly C end was added to cDNA using terminal deoxytransferase and 5' RACE- PCR was performed using primer PolyG forward primer and Dre33Rev reverse primer (5'-GTA CGT ACA TAC GTC CAA CAT A-3'). Obtained products were cloned into pGEM T-Easy Vector and sequenced by Sanger method. Dre-33 cds sequence is 702 bp long and translates into 234 aa. Bioinforamatic analysis confirmed its potential immunogenicity. 80-85% similarity with homologous protein sequences from other filarial worms like *Dirofilaria immitis*, *Acanthocheilonema vitae* or *Brugia malayi* was noted. The similarity to other nematodes like *Toxocara canis* is lower (40-60%). Our results show that Dre33 is a good candidate for a diagnostic antigen for *D. repens* detection.

This study was founded by KNOW (Leading National Research Centre) Scientific Consortium “Healthy Animal – Safe Food”, decision of Ministry of Science and Higher Education No. 05-1/KNOW2/2015.

References

1. Willenbücher J, Höfle W, Lucius R. The filarial antigens Av33/Ov33-3 show striking similarities to the major pepsin inhibitor from *Ascaris suum*. Mol Biochem Parasitol. 1992;57:349-51.

2. Hong XQ, Santiago Meija J, Kumar S, Perler FB, Carlow CKS. Cloning and expression of DiT33 from *Dirofilaria immitis*: a specific and early marker of heartworm infection. Parasitology. 1995;112:331-8.

3. Krushna NSA, Shiny C, Manokaran G, Elango S, Babu S, Narayanan RB. Immune responses to recombinant *Brugia malayi* pepsin inhibitor homolog (Bm-33) in patients with human lymphatic filariaisis. Parasitol Res. 2009;108:407-15.

### P3. The first report of *Wolbachia* spp.in dogs naturally infected with *Dirofilaria* spp. in Serbia - preliminary results

#### Kosić L. S.^1^, Potkonjak A.^1^, Vidanović D.^2^, Kozoderović G.^3^, Vračar V.^1^, Lalošević V.^1^

##### ^1^Department of Veterinary Medicine, Faculty of Agriculture, University of Novi Sad, Serbia; ^2^Veterinary Specialist Institute Kraljevo, Serbia; ^3^-Faculty of Education in Sombor, University of Novi Sad, Serbia

###### **Correspondence:** Kosić L. S. (ljubicask@polj.uns.ac.rs)

Alternative therapy for heartworm disease (HWD) in dogs, consisting of either doxycycline and ivermectin or doxycycline and moxydectine, is widely used in Serbia. The reason for using doxycycline is based on its ability to reduce or remove the burden of *Wolbachia* spp., which exists as an endosymbiont within heartworms. In order to consider whether the usage of doxycycline is justified from this aspect, we retrospectively analyzed the presence of Wolbachia spp. in client-owned dogs with HWD, which had been treated with combined doxycycline and ivermectin. The owners signed a written consent for their dogs to be included in the study. We tested blood samples (whole blood and/or blood sera) of seven dogs infected with *Dirofilaria* spp. (confirmed previously by the modified Knott test for microfilaremia of *D. immitis* and/or *D. repens*, and Canine Heartworm Antigen Test Kit-Snapp HTWM, IDEXX, USA, for circulating antigen of *D. immitis* female) for the presence of *Wolbachia* spp. at the moment of their initial diagnosis of *Dirofilaria* spp., as well as at the end of the alternative therapy (doxycycline and ivermectin). The presence of *Wolbachia* spp. was detected by conventional PCR for wsp gene by using specific primer sets (forward 5'-TGG TCC AAT AAG TGA TGA AGA AAC TAG CTA-3', reverse 5'-AAA ATT AAA CGC TAC TCC AGC TTC TGC AC-3'). At the moment of initial diagnosis of *Dirofilaria* spp. the presence of *Wolbachia* spp. was established in 3 out of 7 dogs. In one dog, *Wolbachia* spp. was present at the end of the alternative therapy for HWD, but not initially. The overall prevalence of *Wolbachia* spp. in dogs infected with *Dirofilaria* spp. was 57.14% (4/7 dogs). At the moment of diagnosis, a high level of *D. immitis* antigen had been detected in three dogs, as well as the presence of microfilariae (microfilariae of *D. immitis* and *D. repens* in two dogs, and microfilariae of *D. immitis* in one dog). The dogs, in which *Wolbachia* spp. was detected at the moment of diagnosis of *D. immitis*, did not have *Wolbachia* spp. after the treatment. It is expected that those dogs were negative for *Wolbachia* spp. due to doxycycline action, which shows the efficacy of treatment. On the other hand, *Wolbachia* spp. was detected in one dog after the treatment, when neither antigen nor microfilariae had previously been detected. Interestingly enough, this dog had had severe HWD (stage 3 with heart failure), but he made a full clinical recovery, and consider cured of HWD when the presence of *Wolbachia* spp. was detected at the end of the therapy. The reason for this might be the desintegration of parasites, a false-negative result for HWD or a new infection during the dog’s treatment. Further studies with improved study design are needed to explain the presence of *Wolbachia* spp. in dogs undergoing the alternative therapy.

### P4. Presence of *Dirofilaria immitis* in dogs and humans in Argentina

#### Gamboa M. I.^1^, Butti M. J.^1^, Radman N. E.^1^, Simón F.^2^, Morchón R.^2^

##### ^1^Chair of Compared Parasitology-Laboratory of Human Parasitoses and Parasitic Zoonoses, School of Veterinary Sciences, National University of La Plata, Argentina; ^2^Laboratory of Parasitology, Animal and human dirofilariosis group, Faculty of Pharmacy, University of Salamanca, Campus Miguel Unamuno s/n, 37007 Salamanca, Spain

###### **Correspondence:** Morchón R. (rmorgar@usal.es)

Cardiopulmonary dirofilariosis (heartworm disease) is a cosmopolitan, serious and potentially fatal disease caused by *Dirofilaria immitis* that mainly affects dogs, while human pulmonary dirofilariosis is a disease that results in small, solitary peripheral pulmonary nodules. Because animal and human dirofilariosis needs the vector activity of culicid mosquitoes for its transmission, adequate environmental factors (moderate/high temperature and humidity) are necessary for both mosquito breeding and the development of infective larvae (L3). The objective of this study was to determine the prevalence of canine dirofilariosis and seroprevalence in humans living in the neighborhood "El Molino", Punta Lara, Ensenada, Buenos Aires, Argentina. Blood samples from 173 dog were analyzed by the in clinic immunochromatographic URANO VET® test, specifically detecting circulating *D. immitis* adult antigens and by a modified Knott technique to detect microfilariae (mf). 79 human samples were analyzed by two laboratory ELISA tests detecting specific anti-*D. immitis* and anti-WSP (*Wolbachia*) IgG antibodies, respectively. Ten (10) out of 173 dog samples (5.7%) presented a URANO VET® positive test and 9 of these contained also mf. Among the 163 URANO VET® negative dogs, 52 (31.9%) contained also mf. In addition, 5 out of the 79 human samples analyzed (6.3%) were positive to both antibody tests against *D. immitis* and WSP. These data reveal the complex situation of the dirofilariosis in Ensenada. The existence of canine microfilaremic infections by *D. immitis* is revealed by the simultaneous positivity to URANO VET® and the Knot tests. Additionally, the absence of mf in a dog positive for the URANO VET® test reveals the presence of some *D. immitis* amicrofilaremic infections. The existence of numerous microfilarmic samples among dogs with negative URANO VET® test suggests the existence of canine filarial species other than *D. immitis*. Similarly, although human infections attributable to *D. immitis* are detected, we can not rule out the existence of zoonotic infections caused by other canine filarial species. More studies are required involving other techniques that allow the unambiguous identification of the mf in each case.

### P5. Proteomic analysis of canine vascular endothelial cells (CnAOEC) and smooth muscle cells (CnAOSMC) stimulated with *Dirofilaria immitis* extracts by LC-MS/MS

#### Morchón R.^1^, González-Miguel J.^2^, Sotillo J.^3^, Carretón E.^4^, Diosdado A.^1^, Zueva T.^1^, Siles-Lucas M.^2^, Montoya J. A.^4^, Simón F.^1^

##### ^1^Laboratory of Parasitology, Animal and human dirofilariosis group, Faculty of Pharmacy, University of Salamanca, Campus Miguel Unamuno s/n, 37007 Salamanca, Spain; ^2^Institute of Natural Resources and Agrobiology of Salamanca (IRNASA-CSIC), 37008 Salamanca, Spain; ^3^Centre for Biodiscovery and Molecular Development of Therapeutics, Building E4, James Cook University, McGregor Rd., Smithfield, Townsville, QLD, 4878, Australia; ^4^Research Institute of Biomedical and Health Sciences (IUIBS), University of Las Palmas de Gran Canaria, 35001 Las Palmas, Spain

###### **Correspondence:** Morchón R. (rmorgar@usal.es)

The cardiopulmonary dirofilariosis caused by *Dirofilaria immitis* is a chronic disease characterized by the development of acute inflammatory reactions and thrombi formation in the pulmonary arteries of the hosts. When spontaneous or treatment induced worm’s death exacerbates inflammatory reactions at the levels of pulmonary arteries, producing a massive release of antigenic products. Nevertheless, adult worms survive for years in immunocompetent hosts. To better understand the influence of worms on the blood vessel endothelium, in the context of the *D. immitis-*host relationships, cultures of canine vascular endothelial cells (CnAOEC) and smooth muscle cells (CnAOSMC) were treated with somatic antigens of *D. immitis* adult worms during the first 24 hours. In both cases unstimulated cells were used as controls. Treated and untreated cells were lysed and analyzed by liquid chromatography and tandem mass spectrometry (LC-MS/MS). As regards CnAOEC, a total of 2573 proteins were identified of which 1308 were in control cells and 1265 in stimulated cells. Of these, 337 (21%) have only been identified in control cells, 294 (18.4%) in stimulated cells and 971 (60.6%) in both. For CnAOSMC, 937 proteins were identified in control cells and 979 in stimulated cells. Of all of them, 224 (18.6%) only appeared in control cells, 266 (22.1%) in stimulated cells and 713 (59.3%) in both. Many of these proteins in previous works have been involved in the parasite-host relationship such as heat-shock proteins, stress response, energy metabolism and redox processes. The differences between some types of cells and others and their similarities are discussed.

### P6. *Dirofilaria immitis* infections in the endangered Iberian lynx (*Lynx pardinus*)

#### Soto L. A.^1^, León-Quinto T.^2^, Bornay Llinares F. J.^1^, Simón F.^3^, Morchón R.^3^

##### ^1^Area of Parasitology, Department of Agrochemistry and Environment, University Miguel Hernández de Elche, Ctra Valencia Km 8.7, San Juan, 03550 Alicante, Spain; ^2^Institute of Bioengineering, University Miguel Hernández, Av. Universidad s/n, 03202 Elche, Spain; ^3^Laboratory of Parasitology, Animal and human dirofilariosis group, Faculty of Pharmacy, University of Salamanca, Campus Miguel Unamuno s/n, 37007 Salamanca, Spain

###### **Correspondence:** Morchón R. (rmorgar@usal.es)

Cardiopulmonary dirofilariosis caused by *Dirofilaria immitis* is a vector-borne parasitic zoonosis. Infections in many species of both domestic (dogs and cats) and wild carnivores (foxes, wolves, coyotes, jackals, raccoon dogs, wild cats and others) have been reported. The complete development of the parasite is mainly supported by the canids, so the importance of other species as reservoirs is discussed [1]. *Wolbachia* are intracellular symbiotic bacteriae exclusively present in some filarial species, including *D. immitis*. In addition to participating in the moult and embryogenesis of filariae, they play an important role in the development of the immune response and immunopathological proccesses associated to dirofilariosis [2]. Information on the prevalence and clinical characteristics of dirofilariosis in wild reservoirs is very limited. In Spain dirofilariosis frequently appears in foxes and sporadically in the wolf [3-5]. Here we investigated the existence of *D. immitis* infections in the endangered Iberian lynx (*Lynx pardinus*), an Iberian endemic species that currently occupies natural restricted and protected areas in Southern Spain or is kept in captivity to proceed with repopulation. All these areas present high or very high risk of Dirofilaria transmission according the geoclimatic prediction model of Simón et al. [6]. Analyses of IgG antibodies anti-*D. immitis* and anti WSPr of *Wolbachia* were carried out in 192 serum samples from animals living in the two main natural protected areas (Sierra Morena and Doñana) and in 3 captive breeding centers, using the ELISA technique. Simultaneous positivity to both tests was used as a criterion to define the existence of infection. Eight (8) out of 192 animals met this criterion (4.1%). Of these, 5 animals lived in free status, 3 in Sierra Morena and 2 in Doñana, while the other 3 were kept in captivity. These data demonstrate the existence of *D. immitis* infections in the Iberian lynx, showing that both free and captive animals are at risk of infection. Complementary studies are necessary to obtain more data that allow us to determine if *D. immitis* infections constitutes a risk for the health and/or life expectancy of the Iberian lynx.

References

1. Simón F, González-Miguel J, Diosdado A, Gómez PJ, Morchón R,. Kartashev, V. The complexity of zoonotic filariasis episystem and its consequences: a multidisciplinary view. Biomed Res Intern Art. 2017:ID 6436130 (In Press).

2. Simón F. Siles-Lucas M, Morchón R, González-Miguel J, Mellado I, Carretón E, Montoya-Alonso JA, et al. Human and aminal dirofilariasis: the emergence of a zoonotic mosaic. Clin Microbiol Rev. 2012;25:507-44.

3. Gortázar Castillo JA, Lucientes J, Blanco JC, Arriolabengoa A, Calvete C. Factors affecting *Dirofilaria immitis* prevalence in red foxes in northeastern Spain. J Wildl Dis. 1994;30:545–7.

4. Mañas S, Ferrer D, Castellá J, López-Martín J. Cardiopulmonary helminth parasites of red foxes (*Vulpes vulpes*) in Catalonia, north-eastern Spain. Vet J. 2005;169:118-20.

5. Segovia JM, Torres J, Miquel J, Llaneza L, Feliu C. Helminths in the wolf, *Canis lupus*, from north-western Spain. J Helminthol. 2001;75:183-92.

6. Simón L, Afonin A., López-Díez LI, González-Miguel J, Morchón R, Carretón E, et al. Geo-environmental model for the prediction of potential transmission risk of *Dirofilaria* in an área with dry climate and extensive irrigated crops. The case of Spain. Vet Parasitol. 2014;200:257-64.

### P7. Elevated levels of eicosanoids are generated during human subcutaneous dirofilariosis

#### García Paniagua R.^1^, Carretón E.^2^, González-Miguel J.^3^, Diosdado A.^1^, Zueva T.^1^, Karsashev V.^4^, Montoya J. A.^2^, Simón F.^1^, Morchón R.^1^

##### ^1^Laboratory of Parasitology, Animal and human dirofilariosis group, Faculty of Pharmacy, University of Salamanca, Campus Miguel Unamuno s/n, 37007 Salamanca, Spain; ^2^Research Institute of Biomedical and Health Sciences (IUIBS), University of Las Palmas de Gran Canaria, 35001 Las Palmas, Spain; ^3^Institute of Natural Resources and Agrobiology of Salamanca (IRNASA-CSIC), 37008 Salamanca, Spain; ^4^Rostov State Medical University, Rostov-na- Donu, 344022, Russia

###### **Correspondence:** Morchón R. (rmorgar@usal.es)

Human subcutaneous dirofilariosis caused by *Dirofilaria repens* is a vector-borne parasitic disease diagnosed with increasing frequency in Europe. These infections are characterized by the appearance of subcutaneous nodules caused by the local inflammatory reaction against immature/mature worms that mimic both benign and malignant primary or metastatic skin tumors [1]. The cells that participate in the inflammatory process synthesize some eicosanoids derived from the arachidonic acid like prostaglandin E2 (PGE2), thromboxane B2 (TxB2) and Leukotriene B4 (LTB4). The aim of this study was to analyze by ELISA the levels of these molecules in serum samples from individuals with human subcutaneous dirofilariosis (G1) and from healthy donors (G2). TxB2 and LTB4 levels were significantly higher in G1 than those obtained by individuals from G2 (P<0.01 and P<0.05, respectively). In addition, levels of TxB2 were always higher than LTB4 levels in both groups. In the case of PGE2, no statistical differences were obtained between groups. These results suggest that elevated levels of TxB2 and LTB4 observed in serum samples from *D. repens* infected patients could be associated with the inflammatory reactions related to nodule formation in human subcutaneous dirofilariosis.

References

1. Simón F, Siles-Lucas M, Morchón R, González-Miguel J, Mellado I, Carretón E, Montoya-Alonso JA. Human and animal dirofilariasis: the emergence of a zoonotic mosaic. Clin Microbiol Rev. 2012;25:507–44.

### P8. Human subcutaneous dirofilariosis in Zamora, Spain: A case report

#### Ramírez de Ocáriz Landaberea I.^1^, Brezmes Valdivieso M. F.^1^, Hernández M. A.^1^, García Sanz S. C.^2^, González-Miguel J.^3^, Simón F.^4^, Morchón R.^4^

##### ^1^Microbiology Unit. Healthcare Complex of Zamora; ^2^Ophthalmology Service. Healthcare Complex of Zamora; ^3^Institute of Natural Resources and Agrobiology of Salamanca (IRNASA-CSIC), 37008 Salamanca, Spain; ^4^Laboratory of Parasitology, Animal and human dirofilariosis group, Faculty of Pharmacy, University of Salamanca, Campus Miguel Unamuno s/n, 37007 Salamanca, Spain

###### **Correspondence:** Morchón R. (rmorgar@usal.es)

Subcutaneous/ocular dirofilariasis caused by *Dirofilaria repens* is a vector-borne zoonosis affecting dogs and humans in the Old World. While dogs are the natural reservoirs, humans can be accidentally infected when they are bitten by mosquitoes that previously fed on infected dogs. Human subcutaneous/ocular dirofilariasis is characterized by the appearance of benign subcutaneous nodules that can be mistaken by cutaneous carcinomas and by the location of immature or fully developed worms free or encapsulated in the ocular area. To the present, in Spain *D. repens* has been detected exclusively in the canine populations of the Mediterranean coastal provinces and Balearic Islands and only 8 human cases have been reported in the same areas. In the present work a clinical case of human subconjunctival dirofilariasis, reported in an area of Northwestern Spain where canine infections by *D. repens* have not been previously observed, is described in a patient with Multiple Endocrine Neoplasia (MEN) syndrome. Surgical removal of the 2 worms with topical anesthesia was difficult as a consequence of the adherence of the parasites to the conjunctiva and their friability. Several fragments of both worms with different sizes were obtained. DNA was extracted, amplified by PCR and visualized in agarose gel electrophoresis. In conclusion, this case should take attention about the possibility to find cases of subcutaneous/ocular dirofilariasis associated to the MEN syndrome and the spreading of *D. repens* into previously non-endemic areas in Spain.

### P9. Epidemiological study on canine filariasis on the border between Lazio and Tuscany (Italy)

#### Sed G.^1^, Morchón R.^2^, Magi M.^1^, Simón F.^2^, Macchioni F.^1^

##### ^1^Department of Veterinary Science, University of Pisa, Viale delle Piagge 2, 56124 Pisa, Italy; ^2^Laboratory of Parasitology, Animal and human dirofilariosis group, Faculty of Pharmacy, University of Salamanca, Campus Miguel Unamuno s/n, 37007 Salamanca, Spain.

###### **Correspondence:** Morchón R. (rmorgar@usal.es)

In Italy the more common filariasis are due to *Dirofilaria immitis*, *D. repens*, and *Dipetalonema* (syn. *Acanthocheilonema*) *reconditum*. While adult worms can reach different anatomical locations depending on the species, the microfilariae are found in the blood or lymphatic vessels. They are vector borne transmitted diseases (mosquitoes, fleas or ticks) and because they possess a very low specificity of definitive host, many species of carnivores can act as reservoirs, of which, domestic and wild canids and felines are of special importance being, in addition, potentially zoonotic. From the clinical point of view, the most important species is *D. immitis* because of the damage it causes to the cardiovascular and pulmonary system, which can pose a serious life risk for infected animals. Canine dirofilariasis is an endemic disease in Italy. *Dirofilaria immitis* presents prevalences ranging from 22 to 80% in Northern areas of the country, while *D. repens* has been observed more frequently in Southern Italy [1]. Recent studies carried out in Central Italy showed a prevalence of 12.5% for *D. immitis* and 12.1% for *D. repens* in Tuscany [2]. In the Lazio region a prevalence of 0.2% for *D. repens* has been observed [3]. The objective of this research is to get information on the prevalence of different filarial species in the canine population a specific area including Northern Lazio (province of Viterbo) and Southern Tuscany (province of Grosseto). Until now, 195 blood samples have been analyzed by Knott and ELISA (Dirocheck) tests: 133 samples from Capalbio (Tuscany) and 62 from Viterbo (Lazio). Prevalences observed were 6.15% for *D. immitis*, 4.62% for *D. repens*, and 0.51% for *Dipetalonema*. These preliminary prevalences are substantially consistent with the data already known for Tuscany, but they open new perspectives regarding the Lazio region, in which very favorable environmental characteristics for the development of vector populations exist. In the future more blood samples from dogs living in these areas will be analyzed, and a study of *A. vasorum*, a species that shares cardiac location with *D. immitis*, will be performed.

References

1. Genchi C, Rinaldi L, Mortarino M, Genchi M, Cringoli G. Climate and *Dirofilaria* infection in Europe. Vet Parasitol. 2009;163:286-92.

2. Magi M, Guardone L, Prati MC, Tozzini G, Torracca B, Monni G, Macchioni F. Canine filarial infections in Tuscany, central Italy. J Helminthol. 2012;86:113-6.

3. Scaramozzino P, Gabrielli S, Di Paolo M, Sala M, Scholl F, Cancrini G. Dog filariosis in the Lazio region (Central Italy): first report on the presence of *Dirofilaria repens*. BMC Inf Dis. 2005;5:75.

### P10. Correlation between selected echocardiographic parameters with optical density after heat treatment in 40 dogs with heartworm disease

#### Tachtsoglou S.^1^, Diakou A.^2^, Polizopoulou Z.^3^, Koutinas C.K.^4^

##### ^1,4^Companion Animal Clinic, School of Veterinary Medicine, Faculty of Health Sciences, Aristotle University of Thessaloniki, Greece, 54627; ^2^Laboratory of Parasitology, School of Veterinary Medicine, Faculty of Health Sciences, Aristotle University of Thessaloniki, Greece, 54627; ^3^Laboratory of Diagnostic Medicine, School of Veterinary Medicine, Faculty of Health Sciences, Aristotle University of Thessaloniki, Greece, 54627

###### **Correspondence:** Koutinas C.K. (mtachtsoglou@gmail.com)

Heartworm disease mainly affects pulmonary arteries, causing intimal proliferation of the occupied vessels and pulmonary thromboembolism by worm fragments. These events commonly lead to pulmonary hypertension (PH). The purpose of this study was to assess the utility of specific echocardiographic parameters to predict worm burden in naturally infected dogs without measuring PH or approximating the number of adult parasites in echocardiography. Forty dogs that had been diagnosed with heartworm disease by antigen and *D. immitis* microfilariae detection or presence of adult parasites in echocardiography, were included in the study. Echocardiography was conducted in all dogs and the following parameters were measured, among others: right pulmonary artery/pulmonary vein ratio (PA/PV), main pulmonary artery/aorta ratio (PA/Ao) and right pulmonary artery/body weight ratio (Radiam/BW). Quantitative serology was performed with DiroCheck (Synbiotics®) after heating the serum at 104 ºC for 10 minutes. Heat treatment of the sera was performed as part of a larger study. The results were quantified by their optical density using the ELISA HUMANREADER photometer reader (HUMAN Diagnostic Systems, Germany®). There was a statically significant positive and moderate correlation when the PA/PV ratio was compared to the optical density after the heat treatment (p<0.002 r=0.461). Furthermore, an equally significant, but low-strength correlation was observed when the PA/Ao ratio was compared to the optical density (p<0.021 r=0.351). Radiam/BW ratio was not significantly correlated to the optical density (p=0.516 r=0.103). The presence of PH is frequent in dogs with heartworm disease but is not always easy to quantify. In addition, it is difficult to gain information about the parasitic burden echocardiographically, by approximating the number of parasites in the right pulmonary artery. This study showed the utility and practical use of PA/PV and PA/Ao ratios for the indirect evaluation of antigen concentration after serum heat treatment, in dogs with heartworm disease.

### P11. Evaluation of serum heat treatment impact on heartworm diagnosis in three groups of dogs with different infection status

#### Diakou A.^1^, Dimzas D.^1^, Tachtsoglou M.^2^, Triantafillou E. A.^3^, Antoniou Κ.^3^, Koutinas C.^2^

##### ^1^Laboratory of Parasitology and Parasitic Diseases, School of Veterinary Medicine, Faculty of Health Sciences, Aristotle University of Thessaloniki, Greece, 54124; ^2^Clinic of Companion Animal Medicine, School of Veterinary Medicine, Faculty of Health Sciences, Aristotle University of Thessaloniki, Greece, 54627; ^3^Private Microbiological Laboratory “VET Analyseis”, Larissa, Greece, 41223

###### **Correspondence:** Diakou A. (diakou@vet.auth.gr)

It has been evidenced that heat pretreatment of serum increases the sensitivity of serological detection of parasitic antigen. This fact has been attributed to the disclosure of the antigen trapped in immune complexes before the treatment. Although heat pretreatment is considered by many a valuable solution for false negative serological results, there is also a concern for false positive results that heat treatment may trigger. The aim of the present study was to evaluate the impact of heat treatment in sera of dogs with different infection status. Three groups of dogs were examined by serology for the detection of *Dirofilaria immitis* antigen. Group 1 (59 dogs) consisted of heartworm infected animals, diagnosed by a snap test or by the Knott’s method, group 2 (26 dogs) consisted of animals living in a non-enzootic area for *D. immitis* and group 3 (94 dogs) included animals of unknown infection status. Heat treatment of the sera was performed at 104^o^C for 10 min. DiroCheck^®^ (SYNBIOTICS) was used in group 1 and FILARCHECK^®^ (biopronix) was used in groups 2 and 3. The optical density (OD) of the results, was measured using ELISA HUMANREADER photometer reader (HUMAN Diagnostic Systems, Germany®). In group 1, 10 animals were seronegative before and only 4 after heat treatment. In group 2 all animals were seronegative before heat treatment and one seroconverted. In group 3, 32 dogs were found seropositive, of which 12 seroconverted after heat treatment. All positive sera before treatment showed increased OD after treatment. Heat-treatment appears to increase the sensitivity of laboratory serological tests, revealing higher concentration of antigen or even a positive result in false negative samples. Heat treatment did not alter significantly the negative result observed in the samples of the non-enzootic area. Furthermore, the animals of group 3 that seroconverted after treatment, were later confirmed as heartworm infected. The results of the present study show that heat pretreatment of sera increases the quantity of antigen circulating in dog’s blood stream, and thus could improve the sensitivity and early diagnosis of heartworm disease.

### P12. Current prevalence of heartworm in stray dogs from Sofia (Bulgaria)

#### Metlarova H., Carretón E., Falcón-Cordón Y., Falcón-Cordón S., Montoya-Alonso J. A.

##### Internal Medicine, Faculty of Veterinary Medicine, Research Institute of Biomedical and Health Sciences (IUIBS), University of Las Palmas de Gran Canaria, 35411, Spain

###### **Correspondence:** Carretón E. (elena.carreton@ulpgc.es)

*Dirofilaria immitis* is a vector-borne parasite and therefore, the transmission depends on the presence of competent vectors, which mainly are different species of culicid mosquitoes. The development and proliferation of mosquitoes depends on climatic conditions like humidity, temperature, and environmental factors. Currently, the climate change, land use modifications due to human activities mainly by agricultural practices or urbanization, as well as the enhanced movement of animal reservoirs and advance of new species of competent mosquitoes, has contributed to the spread of *D. immitis* across Europe. Although is one of the continents where animal dirofilariasis has been studied more widely, some European countries and regions remain scarcely studied. In Bulgaria few studies have been published, reporting the presence of dirofilariasis and gradual increase in dogs and wild canids during the years. To date, the presence of dirofilariasis in dogs in Sofia has been reported, but the prevalence has never been evaluated. Therefore, the aim of this study is to assess the current prevalence and distribution of canine heartworm in dogs from Sofia city and periurban area. Blood samples of 80 adult stray dogs from Sofia and periurban area were collected. Of them, 36 were female and 44 were male; 65 were mixed-bred dogs and 15 were pure-bred dogs. The age range of the dogs went from 1 to 19 years (mean: 7.1 years). All samples were analyzed for circulating *D. immitis* antigens using a commercial immunochromatographic test kit (Urano test Dirofilaria®, Urano Vet SL, Barcelona, Spain) according to manufacturer’s instructions. The prevalence *D. immitis* in in stray dogs in Sofia was 31.25%. Positive dogs were found from 3 to 19 years (mean age: 8.7 years). Prevalence was higher in male dogs (36.4%) compared with females (25%) (P<0.05). No statistically significant differences were observed between breeds. To the authors’ knowledge, this is the first epidemiological report of *D. immitis* in stray dogs in Sofia. Previous studies reported prevalences in stray dogs in different regions of Bulgaria from 12.5% to 33.3%; therefore, the current results are among the highest reported in the country. This could be due to the continuous exposure to the mosquitoes and lack of prophylactic measures against heartworm infection in stray dogs. Sofia is the capital of Bulgaria and also the biggest city of the country, with 1.7 million people living in its metropolitan area. In 2013, it was estimated that 6635 stray dogs lived in the city, being one of the greatest problems of the capital. The present results show that these dogs act as a big reservoir of *D. immitis*. The problem aggravates since the population of Bulgaria is not aware of the prevalence and the importance of this disease. Therefore, a complete epidemiological study of the pet population of Sofia should be carried out to know the current situation of owned-dogs and cats from the city. Also, the results show the need to establish adequate preventive measures for the control of the dirofilariasis as well as awareness campaigns about the severity and importance of this disease for both animals and humans.

### P13. Prevalence of canine and feline heartworm in the Balearic Islands (Spain)

#### Montoya-Alonso J. A.^1^, Carretón E.^1^, Morchón R.^2^, Falcón-Cordón Y.^1^, Falcón-Cordón S.^1^, Simón F.^2^

##### ^1^Internal Medicine, Faculty of Veterinary Medicine, Research Institute of Biomedical and Health Sciences (IUIBS), University of Las Palmas de Gran Canaria, 35411, Spain; ^2^Laboratory of Parasitology, Group of animal and human dirofilariosis, Faculty of Pharmacy, University of Salamanca, Campus Miguel Unamuno s/n, 37007, Spain

###### **Correspondence:** Carretón E. (elena.carreton@ulpgc.es)

The Balearic Islands are an archipelago of Spain located in the western Mediterranean Sea, near the eastern coast of the Iberian Peninsula. They are four largest islands (Mallorca, Menorca, Ibiza and Formentera) and many minor islands and islets close to the larger islands. According to the Köppen-Geiger climate classification, the warm Mediterranean climate (CSa) is the predominant climate. Heartworm (*Dirofilaria immitis*) prevalence has never been assessed in the Balearic Islands, being only reported cases of canine heartworm in Mallorca. The aim of the present work is to evaluate the current canine and feline heartworm status in the three biggest islands: Mallorca, Ibiza and Menorca. Serum samples from 497 dogs and 117 cats presented to veterinary clinics between 2015 and 2016 were analyzed for circulating *D. immitis* antigens using a commercial immunochromatographic test kit (Urano test Dirofilaria®, Urano Vet SL, Barcelona, Spain) according to manufacturer’s instructions. Feline samples were further evaluated by serological techniques for anti-*D. immitis* and anti-Wolbachia antibody detection. General canine prevalence in the Balearic Islands was 3%. By islands, prevalences varied from 0% (Mallorca and Menorca) to 6.5% (Ibiza). All cats were negative for circulating antigens while seropositive cats were only found in Ibiza, showing a seroprevalence of 9.5%. The present study reports the presence of heartworm in pets in the Balearic Islands. Previous report described presence of canine heartworm in Mallorca; however, our results showed no presence of heartworm in that island. The current results show that only Ibiza presents heartworm. Despite the presence of similar climate, presence of microclimates as well as demographic factors and management of pets must be evaluated to determine the heterogeneous distribution of *D. immitis* between islands, similar to observed in the Canary Islands. The results show the need for awareness campaigns and promote the implementation of prophylactic measures in pets in Balearic Islands.

### P14. Heartworm infection in military dogs under preventive treatment: questions and suggested answers

#### Diakou A.^1^, Koutinas C.^2^, Bourguinat C.^3^, Ballesteros C.^3^, Dimzas D.^1^, Chalkias V.^4^, Batra M.^5^. Traversa D.^5^, Prichard R.^3^

##### ^1^Laboratory of Parasitology and Parasitic Diseases, School of Veterinary Medicine, Faculty of Health Sciences, Aristotle University of Thessaloniki, Greece, 54124; ^2^Clinic of Companion Animals, School of Veterinary Medicine, Faculty of Health Sciences, Aristotle University of Thessaloniki, Greece, 546 27; ^3^McGill University, Institute of Parasitology, Sainte-Anne de Bellevue, QC, Canada, H9X3V9; ^4^Military Veterinarian, 350 GMW; ^5^Military Veterinarian, 216 Military Hospital; ^6^Faculty of Veterinary Medicine, Teaching Veterinary Hospital, Teramo, Italy, 64100

###### **Correspondence:** Diakou A. (diakou@vet.auth.gr)

Instances of “loss of efficacy” (LOE) of macrocyclic lactones (ML) in heartworm prevention that have appeared in the US in the past, were in most cases due to a lack of compliance in the administration of preventives. However, in 2014, it was unequivocally proven that ML-resistant heartworm strains were circulating, at least in the Mississippi delta region. In the present study, the investigation of some suspected cases of ML-resistance in military dogs living in Northern Greece, is presented. A military dog living in a camp of Northern Greece with a record of monthly administration of preventives and only one missed dose 2 years earlier, was diagnosed with heartworm disease. Subsequent examination of the remaining 8 dogs of the camp revealed heartworm infection in 4 more animals, with one missed dose of preventives recorded in 2 of them. A few months later, 2 military dogs living in a different camp of Northern Greece were also found heartworm positive, having no recorded missed dose of preventives. In 3 of the infected dogs, microfilariae were counted right before and 7 days after the monthly administration of ivermectin. Furthermore, doxycycline was administered twice (3^rd^ and 6^th^ month of microfilariae count monitoring) for 28 days. Microfilariae from 7 dogs were genetically examined. MiSeq sequencing of regions encompassing 10 single nucleotide polymorphism (SNP) sites, previously identified as highly correlated with ML resistance, was performed and the base frequencies at these SNP positions were extracted using BVA tools. The variance of the allele frequency at a given SNP position was compared to previously described allele frequencies for LOE and susceptible (SUS) populations using Fischer’s Exact test. Level of significance was assessed at p<0.05 and p<0.01. Microfilariae showed a relatively stable count until the 8^th^ month of monitoring, and in most cases less than 75% decrease after each ivermectin administration. However, the genotyping analysis indicated ML-susceptible isolates, thus suggesting that the cause of the heartworm infection was unlikely to be due to drug resistance. All animals of the study were military dogs with expected absolute consistency in preventive treatments. Furthermore, the observations on microfilariae counts after ivermectin challenge at 200 μg/kg, could initially be considered consistent with ML-resistant strains. However, the results of the genetic analysis underline the fact that most cases of suspected lack of ML effectiveness are in reality a result of other factors, such as a temporary lapse in monthly preventative treatment.

### P15. Microfilariemia in the skin capillaries vs. periphereal venous blood in dogs infected with *Dirofilaria* spp

#### Păstrav I. R.^1^, Ionică A. M.^1^, Peștean C.^1^, Novakova E.^2,3^, Modrý D.^3,4^, Mihalca A. D.^1^

##### ^1^University of Agricultural Sciences and Veterinary Medicine Cluj-Napoca, 400372, Cluj-Napoca, Romania; ^2^Biology Centre, Czech Academy of Sciences, Institute of Parasitology, Ceske Budejovice, Czech Republic; ^3^University of South Bohemia, Faculty of Science, Ceske Budejovice, Czech Republic; ^4^University of Veterinary and Pharmaceutical Sciences Brno, Brno, Czech Republic

###### **Correspondence:** Păstrav I. R. (ioana.pastrav@usamvcluj.ro)

Dirofilariases are emerging zoonoses, primarily affecting dogs but also other carnivores. Due to the impact on human and animal health, the transmission of *Dirofilaria* spp. has been vastly studied. The microfilariaemia has been shown to have a circadian variation which may cause difficulties in the screening process. Furthermore, even though mosquitoes feed on capillary blood, the screening is based on peripheral venous blood samples. In this context, the present study aimed to evaluate a capillary sampling method of blood collection using triatomine bugs, to assess the feasibility of measuring the values of microfilariaemia in a dog coinfected with *D. immitis* and *D. repens*, and to evaluate comparatively the circadian variation of capillary vs. peripheral venous blood. One naturally coinfected dog was sampled both in the morning and in the night, using triatomine bugs for capillary blood samples and a syringe for the peripheral venous blood. All samples were processed by modified Knott’s test and morphologicall analysed using a microscope. The results showed variations in the blood meal volume ingested by the bugs, and a feeding success of 50%. The relative values of microfilariaemia were correlated with the blood meal volume: the more blood recovered the higher values of relative microfilariaemia in the evening samples and the opposite for the morning samples. *Dirofilaria immitis* microfilariae were dominant in all the samples, but with significantly higher concentration in the periphereal venous compared with the capillary blood. *Dirofilaria repens* microfilariae showed higher values in the capillary blood samples. Our study showed that triatomine bugs can be used as a model for the collection of capillary blood and study of capillary microfilariaemia in dogs.

### P16. Retrospecitive study on heartworm and microfilarial prevalence in peripheral blood of dogs

#### Stanković I., Novaković K., Medić S.

##### Veterynary laboratory for clinical diagnostic, Serbia, 11000

###### **Correspondence:** K. Novaković (s.medic@vetlab.rs)

Cardiopulmonary Dirofilaria is a vector-transmissible parasitic disease of dogs and other Canidae caused by the nematode *Dirofilaria immitis*, known as the heartworm, whose adult forms inhabit pulmonary arteries. With their presence in the blood vessels they create lesions and reduce their lumen, which results, among other clinical signs, an increase in blood pressure. Because of the vector-host relation in this parasitic disease, constant monitoring of the vectors, prevalence in the population, and the effects of preventive and therapeutic use is necessary. The presence of microfilariae in the samples was diagnosed by a modified Knott test, for which 1 ml of EDTA blood was used and 9 ml of 2% formalin, in 10 ml tubes, centrifuged for 5 minutes at 1500 rpm. After extraction of the supernatant, the sediment was dyed in Loeffler stain and then the coloured sediment was observed on the microscope at a magnification of 100x. Adult forms were diagnosed with an ELISA method in microtiter cups, manufactured by DiroCHEK® Canine Heartworm Antigen Test Kit. During three years, 2015, 2016 and 2017, the samples of canine blood have been tested. Some 1341 samples were tested serologically for presence of *D. immitis* antigen. The 1296 samples of EDTA blood have been screened for presence of microfilariae. In 2015, 190 samples were tested for presence of antigen, where 44 samples (23.2%) have been found positive. In the same year, 194 samples have been tested with modified Knott test, and 28 samples (14.4%) were positive. During 2016, 526 samples were serologically tested, of which 138 (26.2%) samples were positive. From 495 samples tested for presence of microfilariae 62 (12.5%) were found to be positive. During 2017, the percentage of positive serology testing grow up to 28,3%, and modified Knott test prevalence was estimated at 11.2%. Based on evident increase of tests undertaken in our lab, it is obvious that the veterinarians became more alert on presence of cardiopulmonary dirofilariasis in canine population. It is good to know that differential diagnosis list includes this parasitic disease in more and more practicing veterinarians. As a vector disease, many factors could be discussed as influence on increase in percentage of positive tested dogs. The weather conditions, vector distribution or the distribution of tested samples. On the other side, the decrease of positive samples tested for presence of microfilariae confirms that the preventive and therapeutic actions and have positive influence at least on dog to dog transmission way.

### P17. The role of stray dogs in heartworm epidemiology in an urban environment

#### Lagou M.^1^, Andreou D.^2^, Dimzas D.^3^, Koutinas C.^4^, Diakou A.^3^

##### ^1^Undergarduate student, Royal Canin Hellas Scholarship, School of Veterinary Medicine, Faculty of Health Sciences, Aristotle University of Thessaloniki, Greece, 54124; ^2^Undergraduate student, School of Veterinary Medicine, Faculty of Health Sciences, Aristotle University of Thessaloniki, Greece, 54124; ^3^Laboratory of Parasitology and Parasitic Diseases, School of Veterinary Medicine, Faculty of Health Sciences, Aristotle University of Thessaloniki, Greece, 54124; ^4^Clinic of Companion Animal Medicine, School of Veterinary Medicine, Faculty of Health Sciences, Aristotle University of Thessaloniki, Greece, 54627

###### **Correspondence:** Dimzas D. (dimitrissnd@yahoo.gr)

Stray dogs are an important reservoir of *Dirofilaria immitis* in endemic areas. This nematode parasite affects dogs, cats and other carnivores. Most importantly, *D. immitis* may also infect humans and is considered a parasite of great zoonotic importance. The aim of the present study was to investigate the prevalence of heartworm infection in stray dogs in the urban environment of Thessaloniki, located in northern Greece, a highly endemic area. Blood samples were collected from 207 dogs in total, 159 from shelters and 48 from stray animals neutering program. Samples were examined by serology (DiroCheck, SYNBIOTICS^®^, San Diego) and by the modified Knott’s method. Of the 207 animals examined, 41 (19.8%) were found positive for *D. immitis* in at least one of the tests applied (Knott’s method and/or antigen detection). Moreover, in 2 of the examined samples, microfilariae of *Dirofilaria repens* and *Acanthocheilonema reconditum* were found, but not any mixed microfilariae infections were detected. The prevalence of heartworm infection in stray dogs in the urban environment of Thessaloniki was found relatively high. In such environments, stray dogs may play and important role of reservoir and a source of spillover of the parasite to owned dogs. Furthermore, the circulation of the parasite in urban areas increases the risk of human infection, thus may have a great impact in Public Health. Therefore, special care should be given to the populations and health care of stray animals, while preventive measures should be applied to both owned and stray animal, when possible.

### P18. Filarial infections in dogs in Cyprus, an apparently heartworm-free island

#### Dimzas D.^1^, Kokkinos P.^2^, Pantchev N.^3^, Diakou A.^1^

##### ^1^Laboratory of Parasitology and Parasitic Diseases, School of Veterinary Medicine, Faculty of Health Sciences, Aristotle University of Thessaloniki, Greece, 54124; ^2^DVM, Private Practitioner, Cyprus; ^3^IDEXX Laboratories, Germany D-71636

###### **Correspondence:** D. Dimzas (dimitrissnd@yahoo.gr)

Filarial nematodes are common parasites of dogs in south European countries. Among these parasites, the genus *Dirofilaria* includes species of great veterinary and zoonotic importance. Although there is accumulating information about the distribution and prevalence of these parasites in various areas of Europe, relevant information from the island of Cyprus is non-existent. The aim of the present study was to investigate for the first time the prevalence of filarial nematodes in dogs in Cyprus, and to confirm or reject the anecdotal information that this area is heartworm-free. Blood and serum samples were collected between February and August 2017 from 200 dogs from 5 districts of the Republic of Cyprus, i.e. Lefkosia, Lemesos, Larnaka, Pafos and Ammochostos. The animals were at least 12 months-old and were not receiving any kind of preventive treatment for heartworm disease. The samples were examined by the modified Knott’s test and the serological test DiroCheck (SYNBIOTICS^®^, San Diego). The morphological identification of microfilariae was further confirmed by 4 species-specific real-time PCR assays. The target gene for *D. repens* was the cytochrome oxidase subunit I (COI), for *D. immitis* the ITS-1, and for *Acanthocheilonema reconditum* and *A. dracunculoides* the ITS-2. Screening reaction was performed as multiplex PCR and, if positive, 4 separate PCR reactions followed for differentiation. Microfilariae were found in 9 (4.5%) of the animals and were morphologically identified as *A. reconditum*. In all cases, the morphological identification was molecularly confirmed. Furthermore, in the serological examination, 1 (0.5%) sample was positive for *D. immitis* antigen. Unfortunately, it was not possible to confirm this evidence of heartworm infection; however, the animal was not showing any signs of heartworm disease at sampling. Currently, Cyprus can be considered as an apparently heartworm free area. Moreover, no infections with *D. repens* were identified. Thus, it seems that mosquito transmitted filariae are not endemic on the island. However, practitioners should remain vigilant regarding these infections, and consider preventive protection to the animals, at least in case of travel in enzootic areas.

### P19. The case of severe *Dirofilaria repens* infection in a German Shepard dog

#### Klockiewicz M.^1^, Dobrzyński A.^2^, Sobczak-Filipiak M.^3^, Spruch W.^4^, Wysmołek M. E.^1^, Długosz E.^1^

##### ^1^Division of Parasitology and Invasiology, Faculty of Veterinary Medicine, Warsaw University of Life Sciences-SGGW, Ciszewskiego Str. 8, 02-786 Warsaw, Poland; ^2^Department of Small Animal Diseases with Clinic, FVM, WULS-SGGW; ^3^Department of Pathology and Veterinary Diagnostics, FVM, WULS-SGGW; ^4^Veterinary Cabinet – “wwIWet”, Piłsudskiego Al. 38, 05-200 Wołomin, Poland

###### **Correspondence:** Kosic Maciej Klockiewicz (maciej_klockiewicz@sggw.pl)

Sub-cutaneous dirofilariosis in dogs has become quite a common parasitic infection in Warsaw agglomeration. Due to its nature, the diseases may remain undiagnosed for a very long time. The infection is considered particularly dangerous in animals kept outside as guard dogs, because they are continuously exposed to infection transmitted by mosquito vectors. Massively infected animals may develop serious health problems. The 8 year-old, male, German Shepard was reported to the Veterinary Clinic with severe dermatitis of the distal part of the back and pubic area. Deep skin wounds, several centimeters long along both sides of the scrotum were also noticed. General physical condition was estimated as very poor - advanced emaciation and weight loss were noticed. Before surgery, blood test revealed enormously high microfilaremia–14.5x10^3^/1ml of blood. Due to severe status, basic hematological and biochemical blood parameters were monitored for several months. Additionally, HE slides of tumors and skin samples were analyzed because of suspected co-existing atopic dermatitis. During castration massive precipitate was found on the inner side of the scrotum. Investigation of the testicles’ tissues revealed small size tumors containing 26 alive worms and a hard/soft precipitate. After the surgery and normalization of the health status - the targeted treatment against *D. repens* with moxidectin at recommended dose was started. Microfilariae disappeared from bloodstream within 6 days after drug administration. Supportive therapy was also provided to avoid possible life-threatening consequences of massive microfilariae and worm death. HE slides of tumors showed cross-sections of worms or caseous necrotic tissues. Skin HE slides of intradigital areas confirmed chronic dermatitis. And consequently, dog was treated using accurate long-term antibiotic and anti-inflammatory therapy. The dog returned to its normal body weight within 3 months, but skin problems still remained to be resolved after 6 months of antiparasitic treatment. It may be concluded that severely complicated case of skin dirofilariosis was successfully treated with recommended moxidectin therapy. However, veterinarians should consider co-existing disease(s) which may therefore interfere with the final result of the healing process.

### P20. Assessment of a variation of the current adulticide protocol for canine heartworm disease

#### Carretón E.^1^, Falcón-Cordón Y.^1^, Falcón-Cordón S.^1^, Morchón R.^2^, Montoya-Alonso J. A.^1^

##### ^1^Internal Medicine, Faculty of Veterinary Medicine, Research Institute of Biomedical and Health Sciences (IUIBS), University of Las Palmas de Gran Canaria, 35411, Spain; ^2^Laboratory of Parasitology, Group of animal and human dirofilariosis, Faculty of Pharmacy, University of Salamanca, Campus Miguel Unamuno s/n, 37007, Spain

###### **Correspondence:** Elena Carretón (elena.carreton@ulpgc.es)

Heartworm adulticide treatment has been modified with major improvements over the years, and the current treatment presents a better prognosis and greater efficacy. The three-dose method is the current recommended protocol by the European Society of Dirofilariosis and Angiostrongylosis and the American Heartworm Society. Consists on the administration of a single dose of melarsomine dihydrochloride, followed 30 days later by two doses, 24 h apart. This protocol is preceded by 2 to 3 months of monthly macrocyclic lactones, aimed to cover the susceptibility gap between the macrocyclic lactones and melarsomine. Our aim was to evaluate a variation of the adulticide protocol of heartworm disease which consists on the administration of 1 month of macrocyclic lactones instead of 2 to 3 months. Fifteen dogs positive to *D. immitis* antigens were treated at the Veterinary Medicine Service of the University of Las Palmas de Gran Canaria. On day 0, health status was assessed by physical examination and complementary imaging diagnosis. Dogs received a pre-treatment of 30 days with doxycycline (10 mg/kg BID) and ivermectin (6 mcg/kg monthly). On day 30, first dose of melarsomine was administered, followed by a second and third dose on day 60 and 61, respectively. On day 90, dog was examined and discharged. Six months after the last adulticide injection, dogs were evaluated, and antigens test and Knott’s test were performed. All dogs completed the treatment without any severe complication. Six months after the last adulticide injection, the antigens test was negative, all dogs were amicrofilaremic and no presence of adult worms was observed by echocardiography. It is considered that the inefficacy of melarsomine against worms younger than 4 months should be avoided by the previous administration of macrocyclic lactones for 2 to 3 months. This would eliminate larvae younger than 2 months and allow older worms to mature and be susceptible to melarsomine. In our protocol, this susceptibility gap would be covered at the time of the administration of the 2nd and 3rd doses of melarsomine, when the worms non-susceptible to macrocyclic lactones on day 0 would be old enough and susceptible to melarsomine. Furthermore, there is evidence that melarsomine is effective against worms younger than 4 months. This modification of the protocol could allow a faster elimination of the presence of *D. immitis*. Moreover, and a greater compliance by the owners may be achieved by reducing the length of treatment. The results show a potentially valid and effective variation of the currently recommended adulticide protocols.

## Oral communications

### O1. *Angiostrongylus* - spread or no spread?

#### Morgan E. R. (eric.morgan@qub.ac.uk)

##### Queen’s University Belfast, BT9 7BL, UK

The ‘lungworm’ of dogs and foxes, *Angiostrongylus vasorum*, appears to have spread in recent years, with new reports in several countries. In the absence of robust surveillance data, however, it is doubtful whether this spread is real, or the result of increasing awareness and improved diagnostic tests. Post mortem data from foxes in the UK (n=988) support the hypothesis of genuine spread between 2005 and 2015. Overall prevalence increased from 7% to 18% and roughly doubled in most areas, e.g. from 23% to 51% in the south-east, while northern areas previously observed free from infection were recently colonised. Mitochondrial DNA sequence data are also consistent with northward spread rather than emergence from previously undetected foci of infection, and moreover confirm that parasite populations are shared between foxes and dogs. Recent data from Switzerland and Germany also suggest geographic spread within those countries. Given establishment in local fox populations, newly colonised areas are likely to become persistently infected, while patchy distribution is becoming more generalised, justifying call for wider protection of dogs. Reasons for geographic spread of *A. vasorum* in Europe are currently unclear. Both wild definitive hosts (foxes) and suitable intermediate host species (snails and slugs) have long been abundant across the continent and cannot explain recent parasite emergence. Most newly colonised areas were predicted to be climatically suitable for *A. vasorum* based on statistical models of historic distribution, so expansion into areas that already had basically suitable conditions for transmission seems the most likely process in play. Subtle interactions between climate and intermediate host availability might assist and accelerate this spread. For example, increased overwinter survival of slugs in the UK could enable infection of foxes earlier in the year, in turn raising infection pressure for dogs. Ongoing work is shedding light on how interaction between climate and snail biology affects the development and availability of infective *A. vasorum* larvae for dogs, including through effects on host thermal preferences, liberation of larvae from dying hosts, and variation in snail and slug species composition across the rural-urban gradient. These factors could be important to future geographic spread and seasonal patterns of infection risk, although it will take significant further work before understanding is solid enough to underpin recommendations for control. Veterinary practitioners facing unknown local epidemiological situations against a background of parasite spread should initiate routine diagnosis of suspicious cases, for example using point of care tests, and hence build a picture of local hazard on which to base their advice. This is an ongoing need, since the level of risk is liable to change as spread continues. New ways of sharing results of such practice-level surveillance would be useful, as would education of owners around the parasite life-cycle and the risks and signs of infection, to increase their engagement with preventive efforts.

### O2. Canine angiostrongylosis in France: Analysis of risk factors

#### Gossart D.^1^, Desquilbet L.^2^, Rivière J.^3^, Polack B.^1^, Guillot J.^1^

##### ^1^Parasitology, BioPôle Alfort, Département des Sciences Biologiques et Pharmaceutiques, Ecole nationale vétérinaire d’Alfort, France; ^2^Biostatistics and clinical epidemiology, Département des Sciences Biologiques et Pharmaceutiques, Ecole nationale vétérinaire d’Alfort, France; ^3^Epidemiology and contagious diseases, Département des Productions Animales et de Santé Publique, Ecole nationale vétérinaire d’Alfort, France

###### **Correspondence:** Bruno Polack (bruno.polack@vet-alfort.fr)

*Angiostrongylus vasorum* is commonly named “the French heartworm” because the first case of infestation was reported in a dog in Toulouse in 1853 and also because the life-cycle of this nematode was elucidated by Pr Guilhon in the veterinary College of Alfort more than fifty years ago. The aim of the present study was to identify potential risk factors in dogs living in France. A case-control study was carried out on 120 privately-owned dogs. Case dogs (n=30) were defined as dogs that presented clinical signs compatible with angiostrongylosis and for which a positive laboratory test (coprology or snap serology) was obtained. For each case, 3 control dogs were selected. Control dogs were without any clinical signs compatible with angiostrongylosis and were living in the same geographic area as the corresponding case. For each animal, epidemiological data (including the characteristics of the dog, the description of his environment and food, the list of anthelmintic treatments, etc.) were collected through a visit to the veterinary clinics and a phone call to the owners. R Studio® software and the BiostatGV website (www.marne.u707.jussieu.fr) were used for statistical analyses. Univariate analyses included comparisons of proportions, and were tested by using either the Chi-square of Fisher's exact statistical tests. Odds ratios were calculated to quantify the associations between exposures and presence of angiostrongylosis. The univariate analysis revealed a statistically significant association between angiostrongylosis and 9 exposures: the age of the dog (< 2 years), the presence of snails and slugs within the dog’s environment, an unlimited outdoor access, the fact that the dog is fed outside and is not supervised when outside, the presence of toys and dishes in the garden, the dog’s hunting practice, and a low frequency of veterinary consultations and anthelmintic treatments (< once a year). These identified exposures can be hypothesized as potential risk factors for angiostrongylosis in France. A multivariate analysis is warranted to confirm their role in canine angiostrongylosis.

**Acknowledgements**

The authors thank IDEXX laboratory for the help in the selection of veterinary clinics were cases of angiostrongylosis had been observed.

### O3. Seroprevalence of *Angiostrongylus vasorum* in dogs from Greece

#### Angelou A.^1^, Schnyder M.^2^, Schaper R. ^3^, Papadopoulos E.^1^

##### ^1^Laboratory of Parasitology and Parasitic Disease, School of Veterinary Medicine, Faculty of Health Sciences, Aristotle University of Thessaloniki, Greece; ^2^Institute of Parasitology, Vetsuisse Faculty, University of Zurich, Switzerland; ^3^Bayer Animal Health GmbH, Leverkusen, Germany

###### **Correspondence:** Elias Papadopoulos (eliaspap@vet.auth.gr)

The nematode parasites *Angiostrongylus vasorum* live in the pulmonary arteries of dogs and right cardiac ventricle causing severe respiratory disease in dogs [1, 2]. The aim of this study was to investigate the seroprevalence of dogs infected with *Α. vasorum* in the various areas of Greece. For the purpose of this study, 1000 dog serum samples were collected from different parts of the country. Each sample was accompanied with a questionnaire, containing information like gender, age, breed, lifestyle, etc. The examination was carried out using a combination of two in house immunoassays (ELISA) to detect the presence of specific antibodies against *A. vasorum* and the parasite antigen [3]. In total, 16 (1.6%) dogs were found circulating antigens of *A. vasorum* and in 9 (0.9%) only antibodies, whilst in 4 (0.4%) both were simultaneously detected. Antigen detection represents an actual infection while specific antibodies detection represents a parasite exposure (immature worms or parasite free animals due to treatment or natural clearness of the infection with remaining antibodies). The results of this study confirm the presence of *A. vasorum* in different parts of Greece. Therefore, it is important to include angiostrongylosis in the differential diagnosis of cases with cardio-respiratory clinical signs.

**Acknowledgments**

The study was financially supported by Bayer Animal Health GmbH.

References

1. Tarversa D, DI Cesare A, Conboy G. Canine and feline cardiopulmonary parasitic nematodes in Europe: emerging and underestimated. Parasit Vectors. 2010;3:62.

2. Nicolle AP, Chetboul V, Tessier-Vetzel D, Sampedrano CC, Aletti E, Pouchelon JL. Severe pulmonary arterial hypertension due to *Angiostrongylus vasorum* in a dog. Can Vet J. 2006;47:792-5.

3. Schnyder M, Tanner I, Webster P, Barutzki D, Deplaces P. An ELISA for sensitive and specific detection of circulating antigen of *Angiostrongylus vasorum* in srum samples of naturally and experimentally infected dogs. Vet Parasitol. 2011;179:152-8.

### O4. The spread of *Angiostrongylus vasorum* in the Swiss fox population in the last 30 years

#### Gillis-Germitsch N., Schnyder M.

##### Institute of Parasitology, Vetsuisse-Faculty, University of Zurich, 8057 Zurich, Switzerland

###### **Correspondence:** N. Gillis-Germitsch (nina.gillis@uzh.ch)

Foxes and dogs are the main definitive hosts of the cardiopulmonary nematode *Angiostrongylus vasorum*. In Switzerland the very first cases of canine angiostrongylosis occurred in a dog kennel in the 1960s, further cases were reported 40 years after. Since then cases of canine angiostrongylosis were identified with increasing frequency from Switzerland and other European countries. Currently, *A. vasorum* is endemic in dogs in all regions of Switzerland below 700 m asl. Where both, dog and fox populations have been investigated, prevalence in foxes exceeds the one of dogs. The aim of this study was to evaluate the emergence of *A. vasorum* in the Swiss fox population in the last four decades. A total of 3,954 blood samples collected from Swiss foxes between 1986 and 2017 were tested with the ELISAs for detection of circulating *A. vasorum* antigen and specific antibodies against *A. vasorum*. The samples were allocated to 3 areas: north-eastern, south-eastern and western Switzerland, with 2722, 990 and 237 samples respectively. The 32 years collection period was divided into 4 collection timeframes: 1986-1992, 1993-2002, 2003-2012 and 2013-2017. In north-eastern Switzerland 1.9% (25/1343) and 1.7% (14/833) of foxes were antigen-positive in the first two timeframes, respectively. Antigen-positivity increased in the following two decades to 18.3% (17/93) between 2003 and 2012 and to 62.0% (281/453) between 2013 and 2017. In south-eastern Switzerland comparable numbers of foxes were antigen-positive during 1986-1992 and 1993-2002: 7.9% (13/164) and 6.5% (45/691), respectively. Between 2003 and 2012 38.5% (52/135) of foxes were antigen-positive. After 2012 no fox samples were available from this area. From western Switzerland fox samples were available from the timespans 1986-1992 and 2003-2012: in the first time period 1.4% (3/222) and in the second 53.3% (8/15) of foxes were antigen positive. Antibody prevalence was generally lower, due to low sensitivity in foxes. Our results indicate that from 1986-2002 *A. vasorum* prevalence in the Swiss fox population was stable at low levels, and that significant increases occurred since 2003 in all investigated areas. This corresponds to the period in which also *A. vasorum-*positive dogs started to be detected in southern and northern Switzerland. We hypothesize that the increasing number of cases of canine angiostrongylosis in the last two decades is due to a simultaneous increase of *A. vasorum* prevalence in the Swiss fox population, confirming their crucial role as reservoir hosts.

### O5. Feline and canine lungworm in the Netherlands 2011-2018: What do the results from the lab tell us?

#### Dirven M. J.^1,2^, Nijsse E. R.^3^, Broens E. M.^3^

##### ^1^Ictuscordis, Zevenbergschen Hoek, The Netherlands; ^2^Department of Clinical Sciences of Companion Animals, Faculty of Veterinary Medicine, Utrecht University, Utrecht, The Netherlands; ^3^Department of Infectious Diseases and Immunology, Faculty of Veterinary Medicine, Utrecht University, Utrecht, The Netherlands

###### **Correspondence:** M. J. Dirven (mark.dirven@ictuscordis.nl)

Feline and canine lungworm infection in pet cats or dogs may be associated with significant morbidity and mortality. Routine diagnosis usually relies on isolation of first-stage larvae (L1) by larval migration methods (Baermann technique). Reports on the presence of *Aelurostrongylus abstrusus* (feline), *Angiostrongylus vasorum* (canine) and *Crenosoma vulpis* (canine) have been published in the Netherlands. However, infection may be emerging and underdiagnosed. With the help of others, the authors have made an effort in recent years to increase lungworm awareness among veterinary practitioners in the Netherlands. The objective of our study was to report on both the number and the results from Baermann tests from cats and dogs submitted by to the Veterinary Microbiological Diagnostic Centre (VMDC) of Utrecht University from 2011-2017. The results may serve to learn more about the level of awareness, relative prevalence and geographical distribution of feline and canine lungworm in the Netherlands. A database search was conducted to retrieve the total number and the results from Baermann tests from cats and dogs submitted to the VMDC by veterinarians from January 2011 until November 2017. From January 2011 until November 2017 453 faecal samples from cats were examined with the Baermann technique for the presence of lungworm larvae. In 17 samples *A. abstrusus* larvae were found (3.8 %). Positive samples originated from different areas throughout the Netherlands. The total number of samples submitted to the laboratory has increased gradually over the years from 21 in 2011 to 106 in the first ten months of 2017. The number of positive samples has not changed much in the same time period with only a handful of positive samples annually (varying from 0-8 from year to year). From January 2011 until November 2017, 1231 faecal samples from dogs were examined with the Baermann technique for the presence of lungworm larvae. In 23 samples *A. vasorum* larvae were found (1.87 %). Positive samples mostly originated from The Hague and from Flevoland. In 7 samples *C. vulpis* larvae were found (0.56 %). Positive samples originated from different areas than the *A. vasorum* positives. The total number of samples submitted to the laboratory has increased gradually over the years from 114 in 2011 to 279 in the first ten months of 2017. The number of positive samples has not changed much in the same time period ranging from 3-6 positive samples annually. The results from our study suggest that infection with *A. abstrusus, A. vasorum* and *C. vulpis* occurs in cats or dogs in the Netherlands. Feline lungworm seems to be more prevalent than canine lungworm. *A. abstrusus* is found throughout the country. Positive samples for *A. vasorum* seem to originate from two specific areas in the Netherlands whereas positive samples for *C. vulpis* are found in other parts of the country. Local differences may either suggest hyperendemic regions and/or local differences in lungworm awareness, but further research is needed to clarify this. The number of samples submitted by veterinarians for Baermann examination from both cats and dogs has increased gradually over recent years. This most likely reflects increased awareness among veterinarians. In contrast to the total number of submitted samples, the number of positive Baermann tests does not seem to have increased as much. As in other countries, lungworm remains an important differential diagnosis to rule either in or out in any cat or dog with compatible clinical signs.

### O6. Prevalence of metastrongyloid lungworm larvae in Colombian giant African snails (*Achatina fulica*)

#### Lange M. K.^1^, Penagos-Tabares F.^1^, Vélez J.^1,2^, Gutiérrez J.^2^, Hirzmann J.^1^, Chaparro-Gutiérrez J. J.^2^, Taubert A.^1^, Hermosilla C.^1^

##### ^1^Institute of Parasitology, Justus Liebig University, 35392 Giessen, Germany; ^2^CIBAV research group, Veterinary Medicine School, University of Antioquia, 050034 Medellín, Colombia

###### **Correspondence:** M. K. Lange (malin.k.lange@vetmed.uni-giessen.de)

Several metastrongyloid lungworms are considered neglected in Colombia. Whilst *Aelurostrongylus abstrusus* was rarely reported in cat populations, Angiostrongylus vasorum and *A. costaricensis* were occasionally found in Colombian wildlife animals. In contrast, *Crenosoma vulpis* and *Troglostrongylus brevior* have never been detected in the Colombian territory, so far. Research on these lungworm infections is therefore urgently required to gain information on actual prevalences. Since all of these nematodes have terrestrial gastropods as intermediate hosts, we analysed giant neozoan African snails (*Achatina fulica*) from different regions of Colombia for presence of *A. vasorum, A. costaricensis, A. cantonensis, C. vulpis, T. brevior* and *Ae. abstrusus* infections. In total, 609 *Ac. fulica* snails were collected from the following municipalities of Valle del Cauca, Antioquia and Putumayo: Tuluá (Pacific region), Andés, Ciudad Bolívar, Cañasgordas (all Andean region) and Puerto Leguízamo (Amazonian region). Snails were cryo-euthanized, submitted to artificial digestion via pepsin/HCl treatments and examined microscopically for presence of metastrongyloid larvae. Positive samples were confirmed via species-specific PCR followed by sequencing. In total, 9.4 % of *Ac. fulica* proved positive for *Ae. abstrusus*, 3.9 % for *A. vasorum*, 1.3 % for *T. brevior* and 1.1 % for *C. vulpis* larvae. Overall, no infections with *A. costaricenis* and *A. cantonensis* were confirmed via PCR and sequencing. Interestingly, sequence analyses revealed that the current *A. vasorum* specimen belonged to the European lineage, which differs from South American isolates. The current study presents first report on *A. vasorum*, *C. vulpis, T. brevior* and *Ae. abstrusus* infections in neozoan intermediate hosts in Colombia and shows for the first time the presence of the European genotype of *A. vasorum* in South America.

### O7. *Angiostrongylus* species in wild carnivores from Romania: an update

#### Deak G., Ionică A. M., Mihalca A. D., Gherman C. M.

##### Department of Parasitology and Parasitic Diseases, University of Agricultural Sciences and Veterinary Medicine Cluj-Napoca, Romania, 400372

###### **Correspondence:** A. M. Ionică (ionica.angela@usamvcluj.ro)

The genus *Angiostrongylus* (superfamily Metastrongyloidea) comprises 21 species, parasitic in the pulmonary arteries and right heart of a wide variety of mammalian hosts. Among these, three species have been reported in European carnivores, namely *A. vasorum* in wild and domestic canids, *A. chabaudi* in wild and domestic cats and *A. daskalovi* in badgers. The aim of the present study was to provide new insights into the diversity, ecology and distribution of *Angiostrongylus* species in Romanian carnivores. Between February 2015 and March 2018, 788 legally hunted or road-killed carnivores (Canids: 567 red foxes, *Vulpes vulpes*, 87 golden jackals, *Canis aureus*, 14 grey wolves, *C. lupus*; Felids: 26 wildcats, *Felis silvestris*, 4 Eurasian lynxes, *Lynx lynx*; Mustelids: 30 badgers, *Meles meles*, 37 European polects, *Mustela putorius*, 2 steppe polecats, *M. eversmanii*, 12 Eurasian otters, *Lutra lutra*, 7 beech martens, *Martes foina* and 2 pine martens, *M. martes*) were examined by necropsy. All nematodes recovered from the pulmonary arteries and right heart were morphologically identified. Species identification was also confirmed by random specimen sequencing. Overall, 42 animals belonging to three species were positive for *Angiostrongylus* infection. *Angiostrongylus vasorum* was recovered from 24 red foxes (4.2%) originating from the western, north-western and central regions, *A. chabaudi* was found in 7 wildcats (26.9%) originating from the north-western and eastern regions, while *A. daskalovi* was identified in 11 badgers (36.7%) from the western and north-western regions. All other wild carnivores were negative. The present study reveals the occurrence of three *Angiostrongylus* species in Romania’s sylvatic fauna. In the western part of the country, other authors reported larvae morphologically identified as *A. vasorum* in 5.21% (6/115) of examined dog faecal samples. However, no information regarding the clinical status of the infected animals was provided. To the best of our knowledge, so far, no clinical cases have been reported in domestic cats. However, our data strongly suggests the potential emergence of such cases in the near future, therefore an increased surveillance of pet animals would be advisable, particularly in the endemic areas.

### O8. New clinical aspects in dogs infected with *Angiostronylus vasorum*

#### Schnyder M. (manuela.schnyder@uzh.ch)

##### Institute of Parasitology, Vetsuisse-Faculty, University of Zurich, 8057 Zürich, Switzerland

A broad range of clinical signs, not all correlated with classical respiratory distress, has given canine angiostrongylosis the attribute of a “great imitator”. This increases the risk for misinterpretations and delayed diagnosis of the infection, particularly in recently discovered endemic areas. Bleeding diathesis is a potentially lethal manifestation that has been observed in up to one-third of clinical cases. Several altered laboratory parameters and therefore affected steps in the coagulation pathway were described but the exact pathomechanisms are is still unclear. Importantly, hypocoagulation is frequent; this can be followed-up by rotational thromboelastometry (ROTEM), illustrating the changes in viscoelasticity of whole blood under standardised shear conditions. Hypocoagulability and low fibrinogen concentration were previously observed in bleeding dogs infected with *A. vasorum*. In a study with dogs naturally infected with *A. vasorum* that were divided into animals with clinically visible signs of bleedings and without, hyperfibrinolysis was significantly more frequent in bleeding dogs. This was associated with severe hypofibrinogenemia, and hypothesised to represent a relevant mechanism of bleeding diathesis induced by the parasite. Interestingly, survival to discharge was not significantly different between bleeding and non-bleeding dogs: this may have been supported by a novel treatment approach using tranexamic acid (in addition to plasma transfusions), which is an antifibrinolytic drug commonly used in human medicine. *Angiostronylus vasorum* infections were further diagnosed in dogs with e.g. neurological disorders, ocular damage, hepatic abnormalities or dermatitis. Little is known about the impact on kidneys: eggs, larvae, inflammatory cells and haemorrhages were previously observed in the kidneys of different final hosts. In an experimental, 8 weeks after infection renal samples were obtained and fixed for light and electron microscopy analysis: in 2/8 dogs fetal glomeruli and in 3/8 multifocal granulomas were observed in the cortex. No glomerular immune deposits were found. In a comparable group treated with an anthelmintic after 30 days, 3/8 also had occasional fetal glomeruli and one had focal and segmental mesangial sclerosis and occasional mesangial interposition. In the same dogs, IL-2, IL-6, IL-8, IL-10 and TNF-α were measured with dog specific sandwich immunoassays using electrochemiluminiscence. Before the infection there was no significant difference between treated and untreated dogs, while at the end of the study significant differences were observed for IL-8 and IL-10. Regulatory cytokines may contribute to the long term survival of *A. vasorum* in the host: avoiding an increase or even decreasing pro-inflammatory cytokines might be part of the survival strategy of the parasite in dogs.

### O9. First autochthonous clinical case of canine angiostrongylosis in Greece

#### Tachmazidou A.^1^, Papaioannou N.^2^, Diakou A.^3^, Di Cesare A.^4^, Traversa D.^4^, Savvas I.^1^, Patsikas M.^1^, Stylianaki I.^5^, Mylonakis M. E.^1^

##### ^1^Companion Animal Clinic, School of Veterinary Medicine, Faculty of Health Science, Aristotle University of Thessaloniki, Greece, 546 27; ^2^Laboratory of Pathology, School of Veterinary Medicine, Faculty of Health Science, Aristotle University of Thessaloniki, Greece, 54124; ^3^Laboratory of Parasitology and Parasitic Diseases, School of Veterinary Medicine, Faculty of Health Science, Aristotle University of Thessaloniki, Greece, 54124; ^4^Faculty of Veterinary Medicine, University Teaching Veterinary Hospital, Teramo, Italy, 64100; ^5^Laboratory of Pathology, School of Veterinary Medicine, Faculty of Health Science, Aristotle University of Thessaloniki, Greece, 54124

###### **Correspondence:** A. Dialou (diakou@vet.auth.gr)

A 7-month-old, male, mixed-breed, formerly stray and recently adopted dog, living in a yard in the suburbs of Thessaloniki, Northern Greece, was presented with progressively worsening anorexia (4-wk duration), coughing and vomiting during the last week. Clinical examination indicated poor body weight, tachypnea, respiratory distress, tachycardia, pale mucosae, melena and dehydration. Laboratory testing revealed anemia, mild neutrophilic and eosinophilic leukocytosis, mild thrombocytopenia, hyperproteinemia, normal prothrombin and thromboplastin clotting times and increased bleeding time. The dog was negative for heartworm disease, ehrlichiosis, Lyme disease, anaplasmosis and parvovirus (SNAP 4Dx Test^®^, SNAP Parvo Test^®^, IDEXX). Thoracic radiography indicated a diffuse alveolar patter, suggestive of pulmonary bleeding. The animal was hospitalized in the intensive care unit for optimal ventilation support and blood transfusion, but it died two days later. At necropsy gross examination showed haemothorax, jaundice and multifocal nodular lesions in the lungs. Histopathological examination revealed severe granulomatous pneumonia and intraparenchymal parasitic larvae and eggs. The larvae found in the faeces and the bronchoalveolar lavage were morphologically and molecularly (PCR) identified as *Angiostrongylus vasorum*. Serological examination by Angio Detect (IDEXX) was also positive. This report describes the first autochthonous clinical case of canine angiostrongylosis in Greece. Angiostrongylosis should be considered as a major differential in dogs admitted with cough, respiratory distress and abnormal bleeding, even where the parasite is unexpected.

### O10. Pulmonary sonography in *Angiostronylus vasorum* symptomatic dogs

#### Venco L., Formaggini L., De Franco M., Ferrari C., Manfredini S.

##### Clinica veterinaria Lago Maggiore, Arona, Italy

###### **Correspondence:** Luigi Venco (luigivenco@libero.it)

The most common clinical scenario in canine angiostrongylosis is characterized by acute pulmonary symptoms as acute dyspnoea, coughing, haemoptysis and emergency treatment is usually needed. A recent paper described lung sonographic findings in a dog infected by *A. vasorum* as scattered small sub pleural solid nodules with a diameter of few millimeters. Based on this and on the authors’ personal experiences a prospective longitudinal study on lung sonographic appearance of angiostrongylosis in symptomatic dogs was performed for assessing sensitivity and specificity of these findings. Dogs with acute dyspnoea (Grade IV New York Heart Association Score: Dyspnoea at rest) younger than 2 years referred to our hospital underwent first lung sonography by using a high frequency linear or convex transducer lined in intercostal space followed by thoracic radiographs (if possible), Baermann and Ag test. Presence of sub pleural lung nodules and eventually concomitant presence of B lines as thickening of interstitium (suggesting inflammation or hemorrhage) and or lung lobes consolidation were annotated. Between November 2016 and March 2018, 18 privately owned dogs (7 female, 11 male) were included into the study. Ten of them showed sub pleural nodules, in 6 cases with concomitant presence of B lines (Fig 1) and in one with lung lobe consolidation too. All of these dogs yielded positive results on Antigen and/or Baermann test. Dogs with concomitant B lines showed most severe dyspnea, while the only dog with lung consolidation died two hours after sonography In the remaining cases on lung sonography dyspnea, based on completely different findings, was discovered as being due to pleural effusion (rodenticide poisoning 3 cases, trauma 2 cases, piothorax due foreign body 1 case), pneumothorax (1 case) and diaphragmatic hernia (1 case). Lung sonographic findings of canine angiostronylosis in symptomatic dogs under 2 years of age showed high sensitivity and specificity. Presence of B of lines was consistent with more severe symptoms while lung consolidation was related to a poor prognosis. Angiostrongylosis seems to be furthermore the more common causa of severe dyspnea in dogs less than 2-year-old.

**Fig. 1 (abstract O10).**
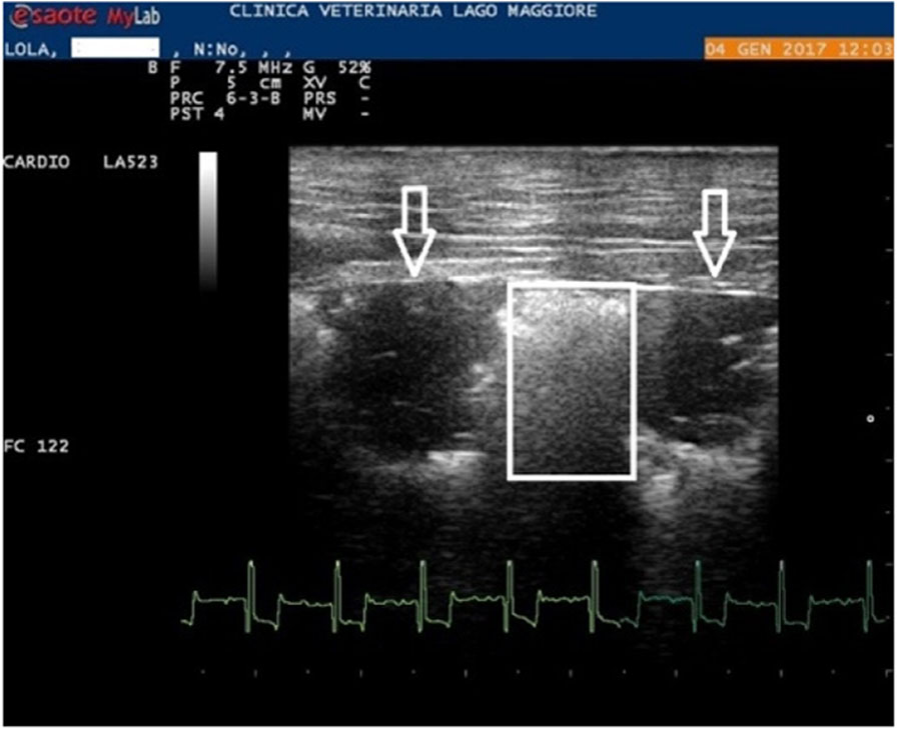
Lung sonography in a 10 month old Maltese dog with severe dyspnea due to *A. vasorum* infection. Sub-pleural nodules (white arrows) and B lines (white square) are shown

References

1. Di Cesare A, Traversa D, Manzocchi S, Meloni S, Grillotti E, Auriemma E, et al. Elusive *Angiostrongylus vasorum* infections. Parasit Vectors. 2015;8:438.
2. Rademacher N, Pariaut R, Pate J, Saelinger C, Kearney MT, Gaschen L. Transthoracic lung ultrasound in normal dogs and dogs with cardiogenic pulmonary edema: a pilot study. Vet Radiol Ultrasound. 2014;55:447-52.

### O11. Serum-protein-electrophoresis in canine angyostronylosis

#### Didier M.^1^, Bertazzolo W.^2^, Gerou-Ferriani M.^1^

##### ^1^Clinica Veterinaria Malpensa, 21017 Samarate, Italy; ^2^MyLav Laboratorio La Vallonea Passirana di Rho, Italy

###### **Correspondence:** M. Didier (martinedidier@hotmail.com)

Dogs infected with *Angiostrongylus vasorum* are reported to have high concentration of globulins, but the electrophoretic pattern has not been deeply evaluated. The aim of this study was: 1) describe the electrophoretic pattern in a group of dogs affected by angiostrongylos. 2) estimate the frequency of this electrophoretic pattern in our laboratory caseload. Cases of angyostrongylosis were collected prospectively between March 2015 and October 2017. *Angiostrongylus vasorum* infection was diagnosed by microscopic identification of larvae after enrichment by the Baermann technique. Serum proteins from these dogs were submitted to capillary electrophoresis to check if a specific migration pattern was present. To evaluate the frequency of the same pattern in our routine caseload, one of the authors (MD) blindly revised all the electrophoresis preformed consecutively in one month in a commercial laboratory. Twenty-one dogs were diagnosed with angiostrongylosis. A specific tracing was observed in 12 cases (57%), consisting of a well-defined peak in the beta-3 region. A total of 1517 serum protein electrophoresis were performed between 2 January 2017 and 31 January 2017. Of these, 20 samples (1.3%) showed a similar peak in the beta-3 region. A specific electrophoretic pattern was found in dogs with angiostrongylosis. The frequency of this pattern was much higher that those observed in a randomly selected canine population.

**Fig. 1 (abstract O11).**
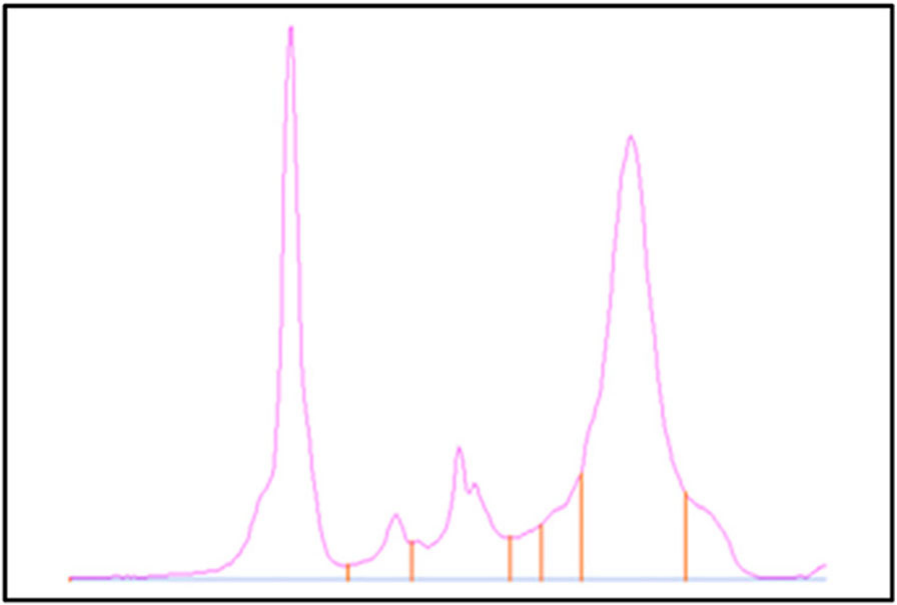
Serum protein electrophoresis from a dog with angiostrongylosis

### O12. Quantitative proteomics analysis of *Angiostrongylus vasorum*-induced alterations in dog serum

#### Tritten L.^1^, Kockmann T.^2^, Schnyder M.^1^

##### ^1^Institute of Parasitology, Vetsuisse-Faculty, University of Zurich, Switzerland, 8057; ^2^Functional Genomics Center Zurich, Switzerland, 8057

###### **Correspondence:** L. Tritten (Lucienne.tritten@uzh.ch)

Blood contains hundreds of proteins, reflecting ongoing cellular processes and immune reactions. Several prior studies identified a perturbed blood protein profile in *A. vasorum*-infected dogs. Some reports describe high levels of total protein, especially of the globulin fraction, however without the necessary depth of analysis in order to resolve the observed pathologies in *A. vasorum* infections, including bleeding disorders. Using sera from 8 experimentally-infected dogs (i) before infection (day -7) with *A. vasorum*, (ii) 34 days post-infection (p.i.; immature infection), and (iii) 75 days p.i. (mature patent infection), serum proteins were measured using liquid chromatography, tandem mass spectrometry (LC-MS/MS). A data-independent acquisition workflow was employed in order to generate quantitative data. Following computational analysis, we identified a number of up- and down-regulated proteins following infection (log2 ratio cutoff ≥ 1.0; q-value ≤ 0.05). Differences in serum profiles were most pronounced at day 75 p.i. compared to before infection. Among up-regulated proteins, chitinase 3, several saposin-like proteins, and heat-shock proteins were found greatly increased (log2 fold-changes ≥ 5). Levels of pulmonary surfactant protein B were elevated on day 34 p.i. already, in the prepatent phase. KEGG pathway “complement and coagulation cascades” was found to be significantly enriched upon analysis of down-regulated proteins. Among them were mannan-binding lectin serine peptidases, ficolin, and coagulation factors. These results reflect the ongoing immune response and stress imposed to the lungs by the parasite. In addition, they bring new elements towards understanding the coagulopathies observed in some *A. vasorum*-infected dogs.

### O13. Clinical pictures in dogs naturally infected with *Angiostrongylus vasorum*

#### di Regalbono F.^1^, Cassini R.^1^, Morelli S.^2^, Grillotti E.^2,6^, Crisi P. E.^2^, Di Giulio E.^3^, De Tommaso C.^4^, Simonato G.^1^, Pezzuto C.^5^, Pampurini F.^7^, Russi I.^2^, Beraldo P.^8^, Viglietti A.^9^, Traversa D.^2^

##### ^1^Department of Animal Medicine, Production and Health, University of Padua, Italy, 35020; ^2^Faculty of Veterinary Medicine, University of Teramo, Italy, 64100; ^3^Ambulatorio Veterinario associato “J. Herriot”, Roseto degli Abruzzi, Italy, 64026; ^4^Labforvet Caserta SAS, Caserta, Italy, 81100; ^5^Ambulatorio Veterinario di Pezzuto Carlo e Piano Noemi, Campobasso, 86100; ^6^Ambulatorio Veterinario Reate, Rieti, Italy, 02100; ^7^Bayer Animal Health, Milan, Italy, 20156; ^8^Division of Animal and Veterinary Science, University of Udine, Udine, Italy, 33100; Ambulatorio Veterinario “Dr. Viglietti-Dr.ssa Piazza”, Carloforte, Italy, 09014

###### **Correspondence:** Frangipane di Regalbono (antonio.frangipane@unipd.it)

In 2015-2018 a study was carried out in various regions of Italy to describe the variability of clinical signs associated with *Angiostrongylus vasorum* in naturally infected dogs. Faecal samples collected from 1050 dogs in nine Italian regions (northern Italy: Veneto, Friuli Venezia Giulia; central Italy: Abruzzo, Marche, Lazio and Sardinia island; southern Italy: Molise, Campania, Puglia) were subjected to the Baermann technique. During clinical examination, epidemiological data of dogs were registered and the prevalence differences in relation to epidemiological data were tested by Chi-square test or Fisher's exact test using the software SPSS Statistics (version 24.0), with a significant level of p<0.01. *Angiostrongylus vasorum* was detected in 84 (8.0%) dogs from Campania (64 dogs), Abruzzo (9 dogs), Lazio (7 dogs) and Molise (4 dogs). Prevalence rates detected in dogs ageing >36 - ≤72, and over 72 months (10.4% and 10.8%), were higher (p<0.01) than in dogs ≤36 months old (4.2%). Significantly higher values of prevalence were detected in crossbred (10.9%) than in purebred dogs (3.7%), and almost all (97.6%) positive animals were privately owned. The prevalence rates of *A. vasorum* were significantly higher in dogs with respiratory signs (23/130, 17.7% vs 61/920, 6.6%), and in those with weight loss (9/33, 27.3% vs 75/1017, 7.4%). Specifically, clinical signs were diarrhea (53.6%), respiratory signs (27.4%), with cough (20.2%) and dyspnea (11.9%) being the most frequent, weight loss (10.7%), haematochezia (6.0%), lethargy (4.8%), exercise intolerance (3.6%), shock (3.6%), neurological signs (2.4%), cyanosis of visible mucosae (2.4%) and coagulopathies (1.2%). Among *A. vasorum*-positive animals, the high occurrence of diarrhea was significantly related to co-infections with intestinal parasites. The present data confirm that angiostrongylosis should be included in the differential diagnosis of any disorder of dogs that can be clinically compatible, even in the presence of non-respiratory signs, that sometimes can be the only findings in infected dogs. Some risk factors can have a role in the epidemiology of angiostrongylosis but it should be kept in mind that *A. vasorum* may infect any dog regardless, age, sex, habitat, breed and lifestyle.

### O14. Field efficacy of Advocate in single or repeated administrations vs *Angiostrongylus vasorum*

#### Traversa D.^1^, Grillotti E.^1,2^, De Tommaso C.^3^, Morelli S.^1^, Crisi P. E.^1^, Di Giulio E.^4^, Pezzuto C.^5^, Venco L.^6^, Pampurini F.^7^

##### ^1^Faculty of Veterinary Medicine, University of Teramo, Italy, 64100; ^2^Ambulatorio Veterinario Reate, Italy, 02100; ^3^Labforvet Caserta SAS, Caserta, Italy, 81100; ^4^Ambulatorio Veterinario associato “J. Herriot”, Roseto degli Abruzzi, Italy, 64026; ^5^Ambulatorio Veterinario di Pezzuto Carlo e Piano Noemi, Italy, 86010; ^6^Clinica Veterinaria Lago Maggiore, Italy, 28040; ^7^Bayer Animal Health, Milano, Italy, 20156

###### **Correspondence:** Donato Traversa (dtraversa@unite.it)

The geographic distribution of *Angiostrongylus vasorum* has recently expanded, with an increasing number of clinical cases throughout Europe, including in areas that were previously free of infection. Clinical pictures of dog angiostrongylosis are variable on an individual basis, and include mainly cardio-respiratory signs, coagulopathies and neurologic disorders. The infection is life-threatening if not promptly diagnosed and treated. The spot-on solution containing imidacloprid 10%/moxidectin 2.5% (Advocate®, Bayer Animal Health) has been marketed a decade ago to treat dog angiostrongylosis in single or repeated administrations. The present study aimed to monitor the field efficacy of Advocate® in treating *A. vasorum* infection in different clinical settings from Italy. A total of 75 naturally infected dogs, 15 from Abruzzo (Site A), Lazio (Site B), Molise (Site C) and 60 from Campania (Site D) region, respectively, were enrolled. After a clinical examination (day 0) a single dose of Advocate® was administered to each dog. Physical examinations and Baermann’s test were performed for all dogs at 28-days intervals and, when still positive, animals received an additional dose of Advocate® until Baermann’s test negativization and clinical recovery. A single dose of Advocate® was 100% effective for all dogs from sites A-C. In Site D 50 dogs received two administrations, at day 0 and +28 (efficacy on site 83%), while a third dose at day +56 was necessary for the remaining 10 dogs (efficacy on site 100%). The overall efficacy of one, two or three Advocate® administrations in suppressing larval shedding was, respectively, 20%, 87% and 100%. Importantly, a complete clinical recovery of dogs occurred in concomitance with the Baermann’s test negativization. These data confirm the efficacy and safety of Advocate® spot on in the treatment of dogs naturally infected with *A. vasorum*. In certain circumstances repeated administrations are necessary to cure the infection, in particular, as for site D, in areas characterized by intense local parasitological pressure, leading to severe infections due to high parasite burden.

## Posters

### P1. Efficacy of Advocate® spot-on against *Angiostrongylus chabaudi* in a naturally infected wild cat (*Felis silvestris silvestris*)

#### Diakou A.^1^, Dimzas D.^1^, Astaras C.^2^, Savvas I.^1^, Neophytos Κ.^3^, Migli D.^4^, Di Cesare A.^5^, Traversa D.^5^

##### ^1^School of Veterinary Medicine, Faculty of Health Sciences, Aristotle University of Thessaloniki, Greece, 54124; ^2^Forest Research Institute, Hellenic Agricultural Organization “DEMETER”, Thessaloniki, Greece, 57006; ^3^Veterinary Clinic, Neo Rysio, Greece, 57001; ^4^School of Biology, Aristotle University of Thessaloniki, Greece, 54124; ^5^Faculty of Veterinary Medicine, Teaching Veterinary Hospital, Teramo, Italy, 64100

###### **Correspondence:** D. Dimzas (dimitrissnd@yahoo.gr)

*Angiostrongylus chabaudi* is a little-known parasitic nematode of the European wildcat (*Felis silvestris silvestris*). It has been thus far found in Italy, Germany, Greece and Romania. Moreover, two non-patent infections have been recorded in the domestic cat (*Felis silvestris catus*) in Italy. This report describes the first attempt of treating angiostrongylosis due to *A. chabaudi* in a naturally infected wildcat. A young male wild cat was accidentally captured in a hen house in Xanthi (northern Greece). The animal showed signs of exhaustion and was referred to a suitable enclosure at the Forest Research Institute in Thessaloniki for recovery. After sedation, the animal was clinically and radiographically examined. Furthermore, faecal samples were subjected to floatation, sedimentation and Baermann tests. After a diagnosis of angiostrongylosis achieved morphologically and molecularly, a spot-on formulation containing imidacloprid 10%/moxidectin 1% (Advocate^®^, Bayer) was administered and a quantitative follow-up measurement of larvae shedding by Baermann method was daily performed. The clinical examination of the wildcat revealed poor body condition (low weight) and abnormal breathing (crackles, wheezes) during thoracic auscultation. The radiographic examination showed severe lesions of bronchoalveolar and interstitial patterns. After the spot-on application the larvae count showed initially a short-term increase, followed by a sharp decrease and eventual total disappearance by day 13. This is the first and apparently successful treatment attempt of patent angiostrongylosis in a wildcat with compatible clinical signs. The evidence that Advocate® may be effective against *A. chabaudi*, could be useful for treating infected wild cats in wildlife hospitals, and probably also domestic cats, in the case *A. chabaudi* infection will spread in domestic felids in the near future, as recently occurred for canine angiostrongylosis.

### P2. Seroprevalence of *Aelurostrongylus abstrusus* in domestic cats from Portugal

#### Alho A. M.^1^, Lopes A. P.^2^, Mesquita J. R.^3,4^, Cordeiro da Silva A^5^, Brancal H^6^, Vilhena H.^7^, Duarte Correia J.^1^, Madeira de Carvalho L.^1^, Cardoso L.^2^, Schnyder M.^8^

##### ^1^CIISA, Faculdade de Medicina Veterinária, Universidade de Lisboa, 1300-477 Lisbon, Portugal; ^2^Department of Veterinary Sciences and Animal and Veterinary Research Centre (CECAV), University of Trás-os-Montes e Alto Douro (UTAD), Portugal; ^3^Escola Superior Agrária de Viseu, Polytechnic Institute of Viseu, Viseu, Portugal; ^4^Epidemiology Research Unit (EPIUnit), Institute of Public Health, Universidade do Porto, Oporto, Portugal; ^5^Instituto de Investigação e Inovação em Saúde, Instituto de Biologia Molecular e Celular, e Departamento de Ciências Biológicas, Faculdade de Farmácia, Universidade do Porto, Oporto, Portugal; ^6^Clínica Veterinária da Covilhã, Quinta das Ferreiras, Boidobra, Covilhã, Portugal; ^7^Baixo Vouga Veterinary Hospital, Águeda, Portugal; ^8^Institute of Parasitology, Vetsuisse Faculty, University of Zurich, 8057 Zurich, Switzerland

###### **Correspondence:** Ana Margarida Alho (margaridaalho@fmv.ulisboa.pt)

Feline aelurostrongylosis is a parasitic lung disease of domestic cats caused by the metastrongyloid nematode *Aelurostrongylus abstrusus*. It is known to have a widespread distribution, reported in most countries in Europe. However, in Portugal, information is patchy and limited to a few studies performed with standard parasitological techniques. Therefore, 1104 sera were collected from apparently healthy (showing no respiratory signs) owned cats which attended veterinary medical centres from mainland Portugal. An ELISA was used for the detection of antibodies against *A. abstrusus*. A total of 35 cats were seropositive (3.2%; 95% confidence interval [CI]: 2.2–4.4)], indicating a previous contact with this parasitic nematode. By district/region, Vila Real registered the highest seroprevalence with 8.0% (16/200; CI: 4.6–12.7), followed by Aveiro/Coimbra with 3.0% (3/101; CI: 0.6–8.4), Lisbon with 2.9% (6/210; CI: 1.1–6.1) and Oporto with 2.0% (10/500; CI: 1.0–3.6). No seropositive results were found in Covilhã (CI: 0.0–8.4) and Algarve (CI: 0.0–7.0) out of the 42 and 51 cats tested, respectively. The highest seroprevalence was detected in the North of Portugal, particularly in Vila Real, a fairly humid region with mild temperatures, climate conditions that may allow a higher occurrence and dispersion of gastropod molluscs, the intermediate hosts of *A. abstrusus*. This mass screening study expands and updates the information on the actual occurrence and distribution of feline aelurostrongylosis in Portugal. Considering the impact of this disease on the health of cats, these results will be vital to raise the awareness of this underestimated lungworm.

### P3. *Angiostrongylus vasorum* infection in a dog: severe clinical findings and irreversible ocular lesions

#### Ciuca L., Meomartino L., Piantedosi D., Cortese L., Lamagna B., Cringoli G., Rinaldi L.

##### Dep. of Veterinary Medicine and Animal Production, University of Naples Federico II, Italy, 80137

###### **Correspondence:** Lavinia Ciuca (lavinia_vet1@yahoo.com)

“Nala” is a ten months year-old female, intact, German Shepherd dog and was referred for apathy and respiratory distress in a private veterinary hospital. For a couple of weeks, the owners complained about the fact that Nala became lethargic and occasional coughing, especially in the evening. Despite continuous antibiotic and mucolytic treatment received, two days before the clinical examination she stopped eating and drinking and refused any movements. Moreover, the owners referred a reduced eyesight. At clinical examination, Nala showed poor general condition, dehydration and dyspnoea. Radiographic examination revealed an increased opacity of the lungs with mixed pattern: alveolar at the right cranial and caudal lobes, bronchial in the peri-hilar regions. A moderate pneumothorax with a radiolucent space between the sternum and the cardiac apex was present. Ophthalmic examination revealed moderate buphthalmos with marked palpebral and bulbar conjunctival hyperemia and scleral injection in both eyes (OU). Ocular ultrasound revealed exudative retinal detachment, lens subluxation and, in the right eye, a vitreal linear foreign body compatible with intraocular parasite. Thorax ultrasonograophy showed in the right hemithorax several non-vascularized subpleural nodules located in the caudodorsal region of the lung, with a diameter of less than one centimeter (Fig. 1). Based on findings obtained, we suspected a parasitic pneumonia. Nala was subjected to faecal examination by FLOTAC to detect *A. vasorum* first-stage larvae. In the same time an antigen test (Angio Detect^TM^) was performed. The dog resulted positive for *A. vasorum* both at serum antigens and larval detection (LPG=324). The therapy included systemic fenbendazole 50 mg/kg SID for 3 weeks associated with prednisolone 0.5 mg/kg, and systemic antibiotics, topic treatment of triamcinolone acetonide, prednisolone acetate1% OU q6h, OU q8h, dorzolamide hydrochloride 2% timolol 0.5% OU q8 h. One week after first examination, Nala showed bilateral retinal atrophy, with increased tapetal reflectivity, loss of retinal blood vessels and optic atrophy. Nala was re-examined 4 weeks post-therapy and was free of A. vasorum but remained with irreversible lesions in the eyes. Another follow-up was performed 4 months later, and the owner reported no further evidence of systemic clinical signs. Increasing awareness of the importance of alternative migratory routes of *A. vasorum* in dogs will improve our current understanding of the diagnosis and clinical follow-up of this parasitic condition. Therefore, veterinary practitioners should include canine angiostrongylosis in the differential diagnosis of ocular diseases.

**Fig. 1 (abstract P3).**
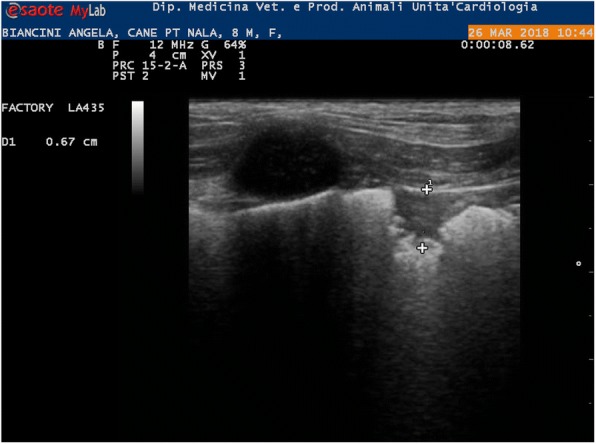
Subpleural nodules located in the caudodorsal region of the lung from a dog with angiostrongylosis

### P4. *Angiostrongylus vasorum* queries submitted to ESCCAP UK & Ireland in 2017

#### Wright I. (hammondia@hotmail.com)

##### Mount Veterinary Practice and ESCCAP UK & Ireland, UK, FY7 6QX

The European Scientific Counsel on Companion Animal Parasites (ESCCAP) UK and Ireland as part of its parasite advice service to vets and the public receives queries via e mail. *Angiostrongylus vasorum* is the subject of many of these queries as the parasite has spread North through the UK over the past two decades and public awareness has been raised through media campaigns. Numbers and type of query regarding a specific parasite are often driven by a lack of familiarity with diagnosis and treatment, or by raised awareness and owner recognition. Here, the types and proportion of *A. vasorum* queries are summarised and the possible causes discussed. Numbers and type of query are recorded by ESCCAP UK & Ireland each year. Queries were divided into parasite distribution/risk of infection, diagnosis, treatment options and prevention. The queries were also calculated as a percentage of the total queries submitted throughout the whole year; 7.1% of 183 queries in 2017 were regarding *A. vasorum*. This was the third highest number of any parasite behind *Leishmania infantum* (10.4%) and fleas (8.4%). The highest numbers of queries were in the summer and autumn with fewest taking place in the winter; 46% of the queries were regarding prevention of *A. vasorum* infection/clinical angiostrongylosis, 30.8% diagnosis and 23.1% distribution in the UK. The relatively large numbers of queries regarding *A. vasorum* is likely a reflection of both its increased distribution and media campaigns to raise awareness. *Leishmania infantum* has seen a steep rise in queries regarding diagnosis and clinical management despite it not being endemic in the UK. This is because of large numbers of cases being seen in imported dogs and Vets in the UK not being familiar with its treatment. Similarly, although *A. vasorum* is endemic, it has spread to new areas where vets will be less familiar with its diagnosis and preventative strategies. The increase in queries of the summer and Autumn may reflect increased prevalence of infection and disease incidence as slug and snail numbers peak but also follows the annual spring media campaign “be lungworm aware”. It is possible that either or both factors are driving interest in the parasite. Increased interest and awareness from vets and the public is to be encouraged as its spread across the UK represents a genuine health risk to dogs, especially where vets and owners may not be prepared to encounter *A. vasorum*. Campaigns such as “be lungworm aware” and parasite awareness/advice groups such as ESCCAP UK & Ireland are vital to both raise awareness and to provide Veterinary professionals with the information they need to protect dogs from the health risks associated with infection.

### P5. Slug intermediate hosts of *Angiostrongylyus vasorum*: vector competence and larval shedding capacity

#### Lange M. K.^1^, Penagos-Tabares F.^1^, Raue K.^2^, Strube C.^2^, Schaper R.^3^, Hermosilla C.^1^, Taubert A.^1^

##### ^1^Institute of Parasitology, Justus Liebig University, 35392 Giessen, Germany; ^2^Institute of Parasitology, University of Veterinary Medicine, 30559 Hannover, Germany; ^3^Bayer Animal Health GmbH, 51373 Leverkusen, Germany

###### **Correspondence:** Malin K. Lange (malin.k.lange@vetmed.uni-giessen.de)

Canine *Angiostrongylus vasorum* infections may cause debilitating disease charcterized by sometimes fatal respiratory, circulatory, bleeding and neurological disorders. The *A. vasorum* life-cycle is obligatory linked to gastropod intermediate hosts being represented by slugs and snails. The main route of canine infection occurs via ingestion of intermediate hosts. Interestingly, gastropod larval shedding into the environment was also reported, thereby representing an alternative route of transmission. Recent European surveys indicate a spread of this parasite and suggest climate-borne shifts in gastropod populations as one potential cause. The current study aimed to elucidate the vector capacity of three most common slug species in Germany (*Arion lusitanicus, Deroceras reticulatum, Limax maximus*) and to evaluate larval shedding by these species. *Arion lusitanicus, D. reticulatum* and *L. maximus* were submitted to dose-dependent experimental *A. vasorum* infections. Therefore, slugs (n = 20) were orally infected with 50 (group A), 200 (group B) and 500 (group C) L1. Every 5 days, two specimens of each group were examined for larvae via artificial digestion. Larval stages were identified morphologically. Furthermore, *A. vasorum*-infected *L. maximus* and *D. reticulatum* were confronted with different stress stimuli (light exposure, heat, dehydration) and analysed for larval shedding in slug faeces and mucus. *Limax maximus* revealed as the slug species with highest infection rate since 30%, 45% and 40% of the specimens were found infected in groups A, B and C, respectively. As expected, high infection rates coincided with high gastropod death rates. *Ar. lusitanicus* showed the lowest infection rates (25%, 15% and 20% in groups A, B and C). Overall, the highest larval burden was found in *D. reticulatum* (100 larvae/slug) followed by *L. maximus* (54 larvae/slug). L2 stages were earliest recorded at day 5 p. i. in *L. maximus* and first L3 stages were found at day 17 p. i. in *Ar. lusitanicus*. Spontaneous shedding of larvae was rare and only observed in three moribund *D. reticulatum* of group B and C. Stimulated larval shedding occurred in up to 85% of the slugs (in both, faeces and mucus) with 1-8 larvae being released per specimen. The three slug species showed different susceptibility to *A. vasorum* infection. Therefore, these species may differentially contribute to the life-cycle of *A. vasorum* in Germany. However, since global warming may favour certain slug species, more research is needed on climate dependency of these intermediate hosts.

### P6. Paratenic transfer of infective third-stage larvae of *Crenosoma vulpis* in experimentally infected gastropods

#### Conboy G., Guselle N.

##### Department of Pathology and Microbiology, Atlantic Veterinary College, Charlottetown, Prince Edward Island, C1A 4P3, Canada

###### **Correspondence:** Gary Conboy (conboy@upei.ca)

A frequent observation from both our field work and laboratory gastropod colony has been that many slug species readily (if not preferentially) consume the tissues of dead slugs (the same or different species). In this study we investigated the potential paratenic spread of metastrongyloid L3 in gastropods due to predation or scavenging by co-housing laboratory-raised slugs (*Limax maximus*) with experimentally infected slugs (*Arion fasciatus*). We then examined whether L3 would survive multiple gastropod-to-gastropod transfers. A total of 80 *A. fasciatus* were collected from a suburban lawn. Twenty were digested in a pepsin-HCL-H2O solution for L3 recovery to determine the presence of natural infection and 3 groups of 20 were exposed to 800, 1600 and 2400 first-stage larvae (L1) of *Crenosoma vulpis*. At 4 weeks post-infection (PI) 4 *A. fasciatus* from each infection group were digested for L3 recovery. At 5 weeks PI, 10 unexposed-laboratory-raised *L. maximus* were placed in each of the 3 infected-*Arion* containers. Surviving *A. fasciatus* from each group were digested after 20 days of co-housing. The co-housed *L. maximus* were digested 4 weeks after the removal of the *A. fasciatus*. The L3 recovered from the co-housed *L. maximus* were placed on lettuce and fed to 36 unexposed *L. maximus* (100 L3/slug). This second group of *L. maximus* was digested for L3 recovery at 391 days PI. No L3 were recovered from natural infection in the 20 digested *A. fasciatus*. After 20 days of co-habitation, 73-80% of the *A. fasciatus* had disappeared without any trace from the 3 containers; 6 of the 30 *L. maximus* had also disappeared. L3 were recovered from all surviving *A. fasciatus*; mean recoveries were 239.2, 268.8 and 387.5 from the groups exposed to 800, 1600, and 2400 L1/slug respectively. L3 were recovered from all surviving co-housed *L. maximus* with mean recoveries of 131.7, 204.7 and 296.7 L3 from the 800, 1600 and 2400 L1/slug infection groups. L3 were recovered from 32/35 (91%) of the slugs fed L3 (Mn = 7.9 L3/slug; Range = 0–26 L3). Paratenic spread of metastrongyloid L3 occurred through the scavenging/ predacious activities of the co-housed *L. maximus* and the L3 were able to survive a second transfer to a third slug. These results indicate metastrongyloid L3 longevity in an ecosystem is probably much greater than previously thought. Further study is required to determine the extent to which paratenic transfer may occur in different species of parasite and slugs, but it is likely to be widespread in both.

### P7. Two case reports of *Angiostrongylus vasorum* in Sardinia, Italy

#### Carta S.^1^, Corda A.^1^, Tamponi C.^1^, Pipia A. P.^1^, Genchi M.^2^, Dessì G.^1^, Manunta L.^1^, Varcasia A.^1^, Scala A.^1^

##### ^1^Laboratorio di Parassitologia, Ospedale Didattico Veterinario, Dipartimento di Medicina Veterinaria, Sassari, Italy, 07100; ^2^Dipartimento di Scienze Medico-Veterinarie, Parma, Italy, 43126

###### **Correspondence:** Marco Genchi (marco.genchi@unipr.it)

*Angiostrongylus vasorum* is a nematode that affects the right heart and pulmonary arteries of dogs and other animals, leading to a chronic condition characterised by cardiac and respiratory failure. The first case reported in a dog in Italy was described by Della Santa et al. (2002) in Tuscany. The present survey describes two case reports of angiostrongylosis in dogs from Sardinia island, Italy. Case 1: A 5 years old, female, neutered, 20 kg, hunting dog was referred for a routine control. The dog did not show any clinical signs. CBC, biochemistry and electrolytes were within the limits except for a hyper γ-globulinemia; thoracic radiography showed a patchy alveolar-interstitial pattern; standard echocardiography did not detect cardiac abnormalities, but the microbubbles test was positive, and it was considered indicative of the presence of pulmonary arteriovenous shunts. L1 larvae of *A. vasorum* were found in faecal sample by Baermann technique and identified based on the characteristic features (wavy tail and cephalic button). DNA was extracted from L1 with a commercial kit (High Pure PCR Template Preparation kit, Roche Diagnostics, Mannheim, Germany) and the diagnosis of angiostrongylosis was confirmed. The dog was treated with Fenbendazole (50 mg/kg bw) for 15 days. Twenty-eight months later the dog did not show any symptoms except for echocardiographic signs of mild PH. No bubbles were observed in the saline contrast echocardiographic study and Baermann technique was negative. Case 2: A two-year-old, male, entire, 18 kg, hunting dog resulted positive during a routine control to the Baermann technique for *A. vasorum*. No clinical signs were present. CBC, biochemistry and electrolytes exams were normal except for a moderate increase in β-globulin fraction. Thoracic radiography showed a patchy alveolar-interstitial pattern; ultrasound displayed no cardiac alterations and the microbubbles test was negative. Diagnosis. Baermann technique revealed the presence of L1 (larvae) of *A. vasorum* with characteristic wavy tail and cephalic button. Moreover, the antigen test (IDEXX Angio Detect™) performed resulted positive to the circulating *A. vasorum* antigen. The patient is currently in therapy with Milbemycin oxyme 0.5 mg/kg per os once a week for 4 weeks. After 2 weeks of treatment it is still positive at Baermann technique. Angio Detect were repeated and resulted positive.

